# Alleviation of a polyglucosan storage disorder by enhancement of autophagic glycogen catabolism

**DOI:** 10.15252/emmm.202114554

**Published:** 2021-09-06

**Authors:** Or Kakhlon, Hilla Vaknin, Kumudesh Mishra, Jeevitha D’Souza, Monzer Marisat, Uri Sprecher, Shane Wald‐Altman, Anna Dukhovny, Yuval Raviv, Benny Da’adoosh, Hamutal Engel, Sandrine Benhamron, Keren Nitzan, Sahar Sweetat, Anna Permyakova, Anat Mordechai, Hasan Orhan Akman, Hanna Rosenmann, Alexander Lossos, Joseph Tam, Berge A. Minassian, Miguel Weil

**Affiliations:** ^1^ Department of Neurology Hadassah‐Hebrew University Medical Center Jerusalem Israel; ^2^ Laboratory for Neurodegenerative Diseases and Personalized Medicine The Cell Screening Facility for Personalized Medicine The Shmunis School of Biomedicine and Cancer Research The George S. Wise Faculty for Life Sciences Sagol School of Neurosciences Tel Aviv University Tel Aviv Israel; ^3^ Blavatnik Center for Drug Discovery Tel Aviv University Tel Aviv Israel; ^4^ Hadassah BrainLabs – National Knowledge Center for Research on Brain Diseases Hadassah‐Hebrew University Medical Center Jerusalem Israel; ^5^ Obesity and Metabolism Laboratory Institute for Drug Research School of Pharmacy Faculty of Medicine The Hebrew University of Jerusalem Jerusalem Israel; ^6^ Department of Neurology Columbia University Medical Center New York New York USA; ^7^ Division of Neurology Department of Pediatrics University of Texas Southwestern Medical Center Dallas TX USA

**Keywords:** adult polyglucosan body disease, autophagy, glycogen, lysosomes, polyglucosan, Autophagy & Cell Death, Genetics, Gene Therapy & Genetic Disease, Organelles

## Abstract

This work employs adult polyglucosan body disease (APBD) models to explore the efficacy and mechanism of action of the polyglucosan‐reducing compound 144DG11. APBD is a glycogen storage disorder (GSD) caused by glycogen branching enzyme (GBE) deficiency causing accumulation of poorly branched glycogen inclusions called polyglucosans. 144DG11 improved survival and motor parameters in a GBE knockin (Gbe^ys/ys^) APBD mouse model. 144DG11 reduced polyglucosan and glycogen in brain, liver, heart, and peripheral nerve. Indirect calorimetry experiments revealed that 144DG11 increases carbohydrate burn at the expense of fat burn, suggesting metabolic mobilization of pathogenic polyglucosan. At the cellular level, 144DG11 increased glycolytic, mitochondrial, and total ATP production. The molecular target of 144DG11 is the lysosomal membrane protein LAMP1, whose interaction with the compound, similar to LAMP1 knockdown, enhanced autolysosomal degradation of glycogen and lysosomal acidification. 144DG11 also enhanced mitochondrial activity and modulated lysosomal features as revealed by bioenergetic, image‐based phenotyping and proteomics analyses. As an effective lysosomal targeting therapy in a GSD model, 144DG11 could be developed into a safe and efficacious glycogen and lysosomal storage disease therapy.

## Graphical abstract

Adult‐onset glycogen storage disorder type 4, or Adult Polyglucosan Body Disease (APBD), is an incurable progressive axonopathy caused by accumulation of poorly branched glycogen inclusions called polyglucosans. Here we test in APBD models the *in vivo*, *ex vivo* and *in vitro* therapeutic efficacy of the new polyglucosan reducing small molecule 144DG11.

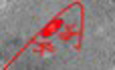

**The paper explained**

**Problem**

Adult polyglucosan body disease (APBD) is an adult onset (˜ 45–50) glycogen storage disorder. APBD is a progressive axonopathic disorder, causing gradual paralysis of the legs. Additionally, APBD is typically characterized by irritable bladder and deficiency of the autonomous nervous system. Average life expectancy of APBD patients is usually 10 years shorter, and death might be caused by recurrent infections due to immobility‐associated pressure ulcers. Currently, APBD is incurable, which generates an unmet medical need. APBD is caused by glycogen branching enzyme (GBE) deficiency leading to poorly branched and therefore insoluble glycogen which precipitates as neuropathogenic polyglucosan (PG) inclusions.

**Results**

We investigated 144DG11, a polyglucosan‐reducing small molecule hit discovered by our previous high‐throughput screening campaign, as a potential APBD therapeutic. As 144DG11 was demonstrated acutely and chronically safe in mice, we tested its efficacy to correct disease phenotypes in the GBE knockin APBD mouse model. We show a strong ameliorative effect of 144DG11 on *in vivo* features compromised by the disease: survival, motor parameters, extension reflex, open field performance, and gait. Importantly, all these ameliorating effects were observed only when 144DG11 was administered two months prior to disease onset, and not after onset, as expected in neurodegenerative disorders, such as APBD, where correction is futile after the death of a non‐renewable mass of neurons. Our results also show that 144DG11 led to a significant reduction in polyglucosan and total glycogen levels in brain, liver, heart, and peripheral nerve, with no apparent effect on muscle PG, in agreement with the pharmacokinetic tissue distribution profile of the compound. As shown in metabolic cages experiments, these reductions ameliorated the otherwise compromised systemic metabolic parameters in GBE knockin mice: 144DG11 increased carbohydrate burn (at the expense of fat burn), respiratory quotient, and total energy expenditure, suggesting metabolic mobilization of pathogenic PG. Interestingly, at the cell level, 144DG11 also increased the relative contribution of glycolytic ATP production at the expense of mitochondrial (OxPhos) ATP production and increased total ATP production. These results suggest that the glucose derived from the enhanced carbohydrate catabolism is exploitable for ATP production. In parallel, we investigated the mechanism of action of 144DG11 *in vitro* and *ex vivo*. Using thermal shift and nematic protein organization technology, validated by surface plasmon resonance and by computational docking simulations, we have discovered that the molecular target of 144DG11 is the lysosomal membrane protein LAMP1 and that its predicted mechanism of action is autolysosomal degradation of glycogen, demonstrated by enhanced autophagic flux and lower levels of lysosomal glycogen. Furthermore, using ratiometric fluorimetry in LAMP1 knockdown APBD fibroblasts, we showed that 144DG11 produces a dominant negative effect which down‐modulates LAMP1 and reduces lysosomal pH, suggesting enhancement of lysosomal degradative function. Finally, to elucidate the functional implications of 144DG11 in APBD cells, we applied image‐based multiparametric phenotyping analysis of APBD patient skin fibroblasts, which revealed that the cell features most ameliorated by 144DG11 are mitochondrial function and lysosomes, which were reduced in size. These results were corroborated by proteomics analysis.

**Impact**

This comprehensive investigation integrating mechanistic and *in vivo* studies lays the groundwork for clinical use of 144DG11 in treating APBD patients who currently have no therapeutic alternative. Moreover, it positions 144DG11 as a lead compound for treating other glycogen storage diseases through safe reduction of glycogen surcharge.

## Introduction

Adult polyglucosan body disease (APBD) is a glycogen storage disorder (GSD), which manifests as a debilitating and fatal progressive axonopathic leukodystrophy from the age of 45 to 50 (Akman *et al*, 2009; Mochel *et al*, 2012). APBD is further characterized by peripheral neuropathy, dysautonomia, urinary incontinence, and occasionally dementia, all being important diagnostic criteria for this commonly misdiagnosed (Hellmann *et al*, 2015; Schwartz *et al*, 2020) and widely heterogeneous disease. APBD is caused by glycogen branching enzyme (GBE) deficiency leading to poorly branched and therefore insoluble glycogen (polyglucosans, PG), which precipitate, aggregate, and accumulate into PG bodies (PB). Being out of solution and aggregated, PB cannot be digested by glycogen phosphorylase. The amassing aggregates lead to liver failure and death in childhood (Andersen’s disease; GSD type IV). Milder mutations of GBE, such as p.Y329S in APBD, lead to smaller PB, which do not disturb hepatocytes and most other cell types, merely accumulating in the sides of cells. In neurons and astrocytes, however, over time PB plug the tight confines of axons and processes and lead to APBD.

The majority of APBD patients are Ashkenazi Jewish bearing the p.Y329S mutation in the *Gbe1* gene (similar to the knockin APBD modeling mice we use here (Orhan Akman *et al*, 2015)). More than 200 confirmed APBD cases worldwide have been reported to date. However, due to common misdiagnosis, this is probably an underestimation. In addition, APBD’s carrier frequency is relatively high (1/58 deduced from testing 2,776 individuals self‐reported to be 100% Ashkenazi Jewish (Akler *et al*, 2020; Schwartz *et al*, 2020), *cf*. 1/27‐1/30 carrier frequency in Tay Sachs disease), and therefore, its prevalence is probably underestimated. Moreover, awareness for the disease among physicians and neurologists has only developed in the last decade.

While an effective cure for APBD is urgently needed, APBD is representative of the larger group of GSDs. GSDs are a varied group of 15 incurable diseases with a combined frequency of 1 in 20,000‐43,000 (Ozen, 2007). Ranging from childhood liver disorders such as GSD1, through adolescent myoclonic epilepsies such as Lafora Disease (LD), and adult progressive neurodegenerative disorders such as APBD, all GSDs are currently incurable. A notable exception is Pompe disease, for which acid alpha‐glucosidase enzyme replacement therapy (Winkel *et al*, 2004; Nicolino *et al*, 2009) and recently the more advanced antibody‐enzyme fusion therapy (Program & Abstracts WORLDSymposium, 2019* 15th Annual Research Meeting, 2019; Zhou *et al*, 2019) are promising approaches, albeit with some immunological complications.

Since all GSDs share the etiology of excessive normal or malconstructed glycogen, we believe that APBD represents a prototypical GSD and thus development of pharmaceutical inhibitors of glycogen accumulation is likely to benefit all GSDs (except for GSD0, where glycogen deficiency, rather than surplus, is considered the pathogenic factor (Orho *et al*, 1998)). Thus, based on the premise that glycogen, or insoluble glycogen in the case of APBD, is a pathogenic factor in GSDs, we have developed an APBD patient cell‐based assay to identify small molecule inhibitors of accumulation of insoluble glycogen or PB (Solmesky *et al*, 2017). Applying this assay in a high‐throughput format, we have screened FDA approved and de novo synthesized compound libraries to discover PB reducing hits, which were further investigated *in vitro* and *in vivo*. One of these hits, the FDA approved glycogen synthase inhibitor guaiacol, indeed demonstrated *in vivo* safety and partial efficacy in the Gbe knockin mouse model of APBD Gbe^ys/ys^ (Kakhlon *et al*, 2018).

Here, we describe the development of a more promising glycogen lowering high‐throughput screen (HTS) hit, 144DG11. Compared to guaiacol, whose main therapeutic effect besides polyglucosan lowering was lifespan extension, 144DG11 also improved motor parameters and carbohydrate burn in Gbe^ys/ys^ mice. Furthermore, our extensive cell and *in vitro* analyses show that 144DG11 targets the lysosomal protein LAMP1 and thus enhances glycophagy and respiratory and glycolytic ATP production, transforming the adversely excessive glycogen into a fuel source. In summary, this study uncovers a new safe and efficacious agent for ameliorating APBD and potentially lysosomal storage disorders and other GSDs instigated by glycogen over‐accumulation.

## Results

### The safe compound 144DG11 improves survival and motor deficiencies in Gbe^ys/ys^ mice

**Figure 1:**
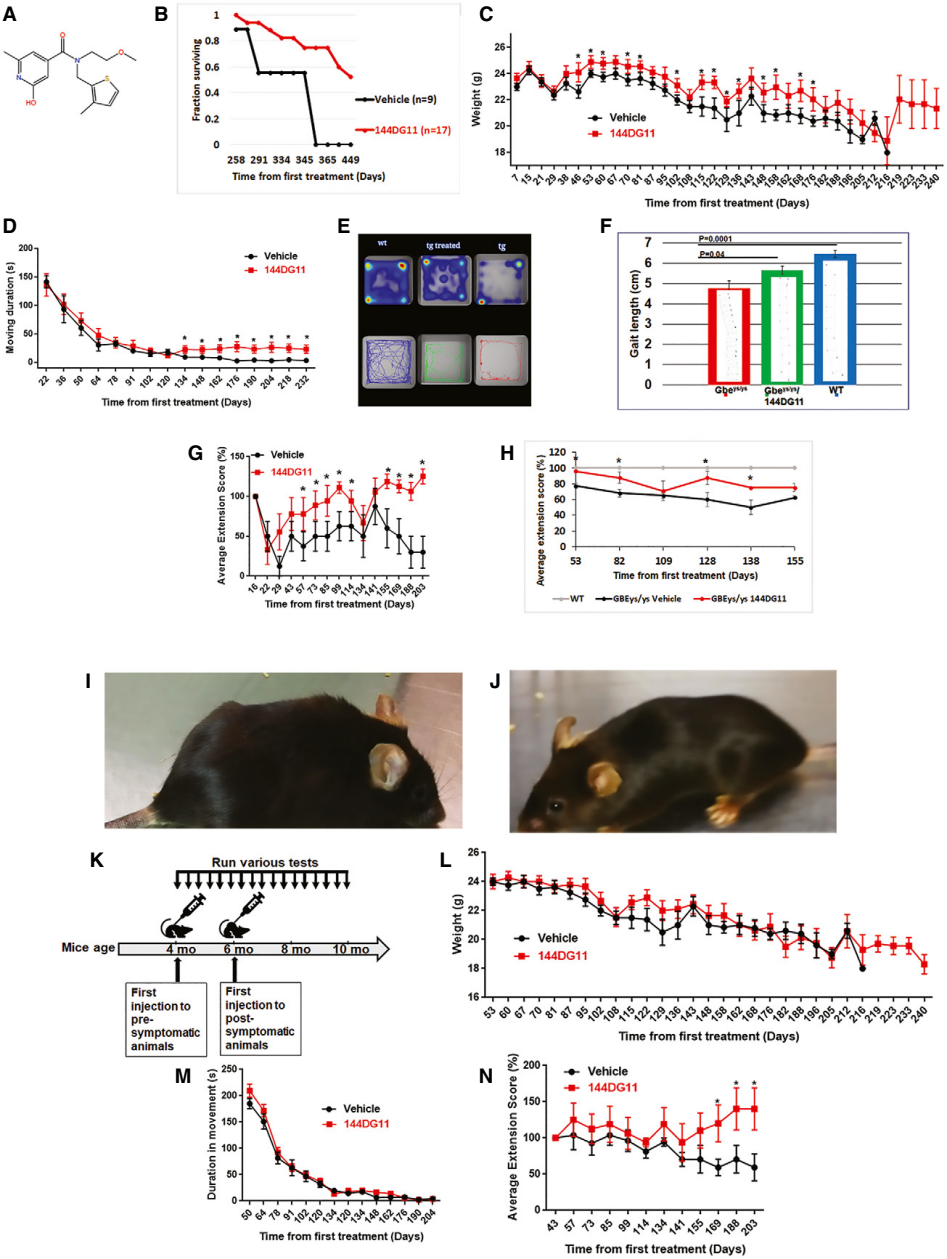
*In vivo* effects of 144DG11 AStructural formula of 144DG11BKaplan‐Meier survival curve based on *n* = 17 144DG11‐treated and *n* = 9 vehicle‐treated animals. 144DG11 significantly increased survival (log‐rank test *P*‐value < 0.000692).C, DWeight and (D) average duration in movement in an open field as a function of time after treating Gbe^ys/ys^ mice with vehicle (*n* = 8), or 144DG11 (*n* = 9).E
*Upper panel*, time spent at location (average based on *n* = 9 nine‐month‐old mice) is quantified as a heat map (blue (low) to red (high) scale). *Lower panel*, visual tracking examples from single animals. wt, vehicle‐treated wt animals; tg treated, 144DG11‐treated Gbe^ys/ys^ mice; tg, vehicle‐treated Gbe^ys/ys^ mice.FGait analysis of *n* = 9 nine‐month‐old mice treated with vehicle or 144DG11. Shown are average (± s.d.) stride lengths. A representative ink foot‐print trail is also shown for each arm. Statistically significant differences (one‐way ANOVA with Sidak’s post hoc tests) were demonstrated between 144DG11 and vehicle‐treated transgenic mice, and between transgenic and wt mice.G, HExtension reflex as a function of time after treating *Gbe^ys/ys^* mice with vehicle (*n* = 8), or 144DG11 (*n* = 9) (G), and, in another experiment (H), *Gbe^ys/ys^* with vehicle (*n* = 8), or 144DG11 (*n* = 9), or wt mice with vehicle (*n* = 8).I, J(I) (vehicle) and (J) (144DG11) show 7‐month‐old Gbe^ys/ys^ mice following 3 months under the respective treatments. Note unkempt fur in vehicle‐treated animal, which was improved by 144DG11.K(K) Experimental design.L–N(L) weight, (M) average duration in movement in an open field, and (N) extension reflex as a function of time after treating Gbe^ys/ys^ mice with vehicle (*n* = 8) or 144DG11 (*n* = 9) at 6 months of age (at onset). Data information: Two‐way ANOVA with repeated measures show that, throughout the period, treatment values were higher than vehicle in (C) (*P* < 0.05), (D) (*P* < 0.1), (G) (*P* < 0.05), (H) (*P* < 0.05), and (N) (*P* < 0.07), but not different than vehicle in (L) and (M) (*P* < 0.15). Significant difference (*P* < 0.05, two‐tailed t‐tests) at specific time points in (C), (D), (G), (H), and (N) is denoted by *. All error bars represent s.d.

**Figure EV1:**
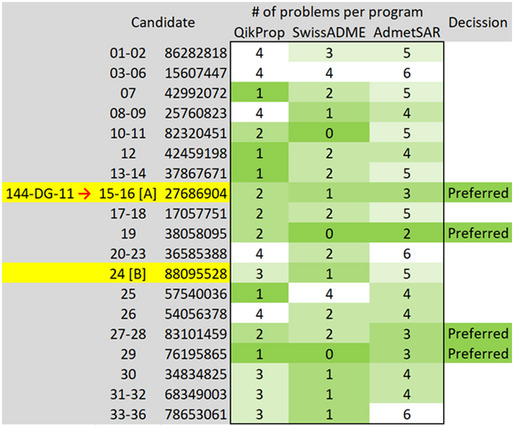
*In silico* ADMET (Absorption, Distribution, Metabolism, and Excretion Toxicity)‐compatible, polyglucosan lowering compounds Heatmap shows analysis of three different ADMET algorithms. On the left are ordinal numbers of the compounds according to the hits discovered in Solmesky *et al* (2017). A range of numbers refers to enantiomers. The second column from left is the ChemBridge catalog number of the compound, and the heatmap (level of green) demonstrates the number of violations predicted by each algorithm.

**Figure EV2:**
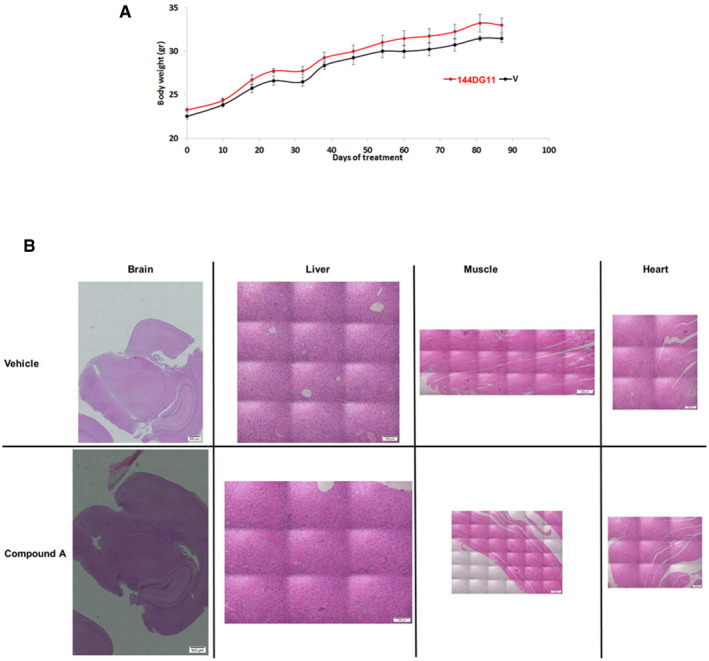
Safety of 144DG11 in mice Body weights of wild‐type C57Bl6J mice treated with 144DG11 for 3 months. Mice were injected twice a week with 150 μl of 144DG11 at 250 mg/kg in 5% DMSO (red, *n* = 5), or an equal volume of 5% DMSO (V, vehicle) control (black, *n* = 6). Injections were intravenous for the first month and then subcutaneous for the following 2 months. No significant change between the two treatments is observed. Error bars represent S.D.Histology of 144DG11 tissues compared to vehicle control. Brain, liver, skeletal muscle, and heart slices of wild‐type C57Bl6J mice treated for 3 months with 144DG11 as in (A). The slices were stained by H&E staining in order to visualize lesions. No lesions were apparent in either treatment. Scale bars, 500 µm (brain), 100 µm (liver), 200 µm (muscle), 100 µm (heart).

We tested 144DG11 (Fig 1A) for its capacity to correct the deficient motor phenotypes and short lifespan in the APBD mouse model Gbe^ys/ys^. 144DG11 is one of 19 PG reducing HTS hits previously discovered by us (Solmesky *et al*, 2017). It was shown by in silico ADMET (Absorption, Distribution, Metabolism, Excretion, and Toxicity) studies to be safe and pharmacokinetically and pharmacodynamically preferred and is therefore worth further pursuit (Fig EV1). Indeed, low ADMET scoring compounds such as “B” (Fig EV1) were not efficacious and caused adverse effects such as wounds (Appendix Fig S1). Moreover, safety assessment in wild‐type (wt) mice confirmed that, administered for 3 months at 250 mg/kg in 5% DMSO (the highest dose possible due to solubility and DMSO toxicity issues), 144DG11 did not influence animals’ weight gain over time (Fig EV2A). The compound also did not produce any histopathological damage or lesions in brain, liver, skeletal muscle, and heart after 3 month exposure (Fig EV2B). Following 1 and 24 h treatments, mice were also examined for abnormal spontaneous behavior, such as immobility, excessive running, stereotyped movements, and abnormal posture (Irwin tests). 144DG11 did not cause any adverse effect in these Irwin tests (Appendix Table S1).

We studied the effects of 144DG11 on motor parameters by administrating the compound at the age of 4 months, two months prior to disease onset, assuming a preferred prophylactic effect. Such an effect is expected in a neurodegenerative disorder such as APBD in which the already dead neurons cannot be affected by a post‐onset treatment. Importantly, as Fig 1B shows, treatment with 144DG11 significantly improved animal survival (log‐rank test *P*‐value < 0.000692) compared to vehicle‐treated animals. Lifespan extension probably mirrors improvement of several parameters related to ability to thrive. The most prominent parameter in that respect is weight. 144DG11 indeed mitigated the decline in animal weight over time caused by the disease (Fig 1C). We also tested the effect of 144DG11 on various motor parameters. 144DG11 improved open field performance (Fig 1D) from a relatively advanced stage of disease progression (8 months, 134 days postinjection (Fig 1D)). These improvements were manifested as increased locomotion and an increased tendency to move toward the center (Fig 1E), perhaps also associated with amelioration of stress and anxiety. The progressive deterioration of Gbe^ys/ys^ mice in open field performance is related to their gait deficiency. Therefore, we tested the effect of 144DG11 on gait at the age of 9 months when gait is severely affected. At that age, 144DG11 indeed improved gait or increased stride length (Fig 1F). Our data also show that, of all motor parameters tested, the most pronounced ameliorating effect was on the overall extension reflex (Fig 1G and H). Overall extension reflex throughout the study period was significantly improved by 144DG11 (Fig 1G, *P* < 0.05) as it was at 9 specific time points (asterisks in Fig 1G). This effect is especially important since its human patient correlate is pyramidal tetraparesis, or upper motor neuron signs, which are one of the main neurological deficiencies in APBD (Mochel *et al*, 2012). The overall beneficial effect of 144DG11 can be best appreciated by Movie EV1 and animal photographs which illustrate that treated animals are less kyphotic and better kempt (Fig 1I and J). Importantly, while open field performance (Fig 1E), gait (Fig 1F) and extension reflex (Fig 1G and H) were significantly improved by 144DG11, they were not restored to wt levels, demonstrating that while efficacious, 144DG11 performance still leaves some room for future improvement.

To validate the assumption that 144DG11 has a prophylactic effect, we also tested the effect of post‐onset administration of 144DG11 on all the parameters improved by 144DG11. The experimental design of pre‐ and post‐onset 144DG11 administrations is shown in Fig 1K. Please note that in post‐onset 144DG11 administration, time from first treatment, which refers to the pre‐onset first treatment, is delayed (compare Fig 1C and D, and G with Fig 1L–N). Weight (Fig 1L), open field (Fig 1M), and *overall* extension reflex (Fig 1N) indeed were not significantly ameliorated when 144DG11 was administered after disease onset at the age 6 months. Notably, extension reflex, the parameter most affected by 144DG11, was also the only parameter improved by the compound from the advanced stage of the disease at the age of 9 months (Fig 1N).

### 144DG11 reduces histopathological accumulation of polyglucosans and glycogen in accordance with its biodistribution

**Figure 2:**
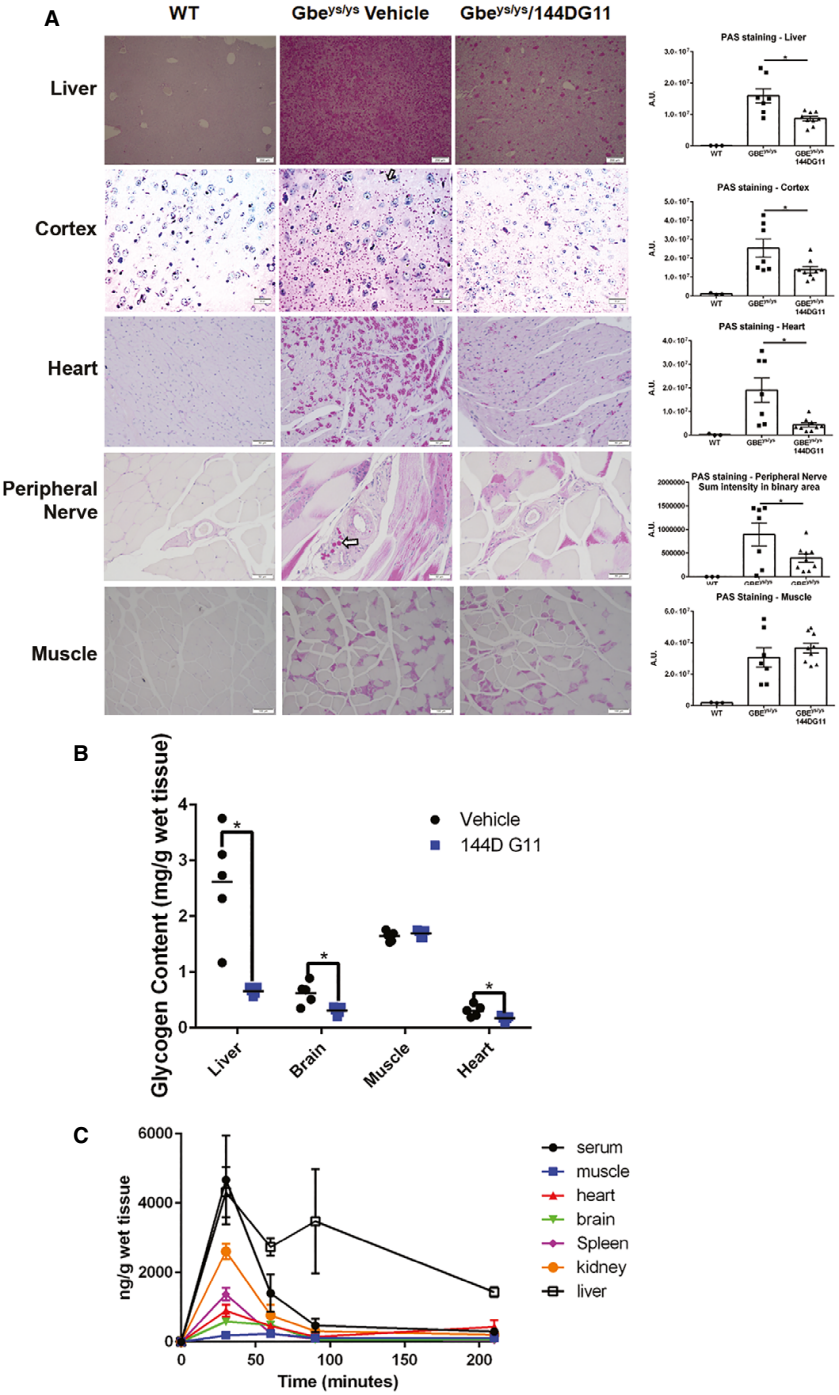
Histopathological effects of 144DG11 and its pharmacokinetics *Left panel*, Mice, treated as indicated, were sacrificed, and the indicated tissues were collected and stained for PG (arrows) with PAS following diastase treatment. Scale bars, 200 µm (liver, muscle), 50 µm (cortex, heart, peripheral nerve). *Right panel*, PAS staining was quantified, as described in (Kakhlon *et al*, 2018), based on analysis of 4 sections from each tissue in *n* = 3 wt, *n* = 7 Gbe^ys/ys^ vehicle‐treated, and *n* = 9 144DG11‐treated mice.Total glycogen in the indicated tissues, collected from *n* = 5 nine‐month‐old vehicle and 144DG11‐treated mice, was quantified as described in (Kakhlon *et al*, 2018). Repeated‐measures 2‐way ANOVA tests show that the pharmacokinetic profile of each tissue is significantly different from that of all other tissues (*P* < 0.05). *, Significant difference (*P* < 0.05) as determined by two‐tailed t‐tests.144DG11 Pharmacokinetics. Gbe^ys/ys^ mice injected with 144DG1 were sacrificed 30, 60, 90, and 210 min postinjection, and the indicated tissues were removed, as well as 200 uL of serum drawn. Graph shows means (± SEM) of 144DG11 levels in the different tissues determined by LC‐MS/MS (see Materials & Methods). Results obtained from *n* = 3 mice at each time point.

As 144DG11 significantly improved motor and survival parameters, we set out to investigate its histopathological effects. This information is important for determining whether the expected mode of action of 144DG11 discovered *ex vivo*—reduction of polyglucosan levels in fibroblasts (Solmesky *et al*, 2017)—also takes place *in vivo* and if so in which tissues. Brain, heart, muscle, nerve fascicles (peripheral nerves), and liver tissues from 144DG11 and vehicle‐treated animals were collected following animal sacrificing at age 9.5 months after pre‐onset 144DG11 treatment from the age of 4 months (Figs 1C and D, and G). The same tissues from wt mice were used as controls. Following diastase treatment to digest non‐polyglucosan glycogen, leaving behind polyglucosan, sections were stained for polyglucosan with periodic acid‐Schiff's (PAS) reagent, counterstained with hematoxylin, and analyzed by light microscopy. The results (Fig 2A) show a significant reduction in polyglucosan levels in brain, liver, heart, and peripheral nerve, with no apparent effect on muscle polyglucosans. Total glycogen levels, determined biochemically as described in (Kakhlon *et al*, 2018), were also correspondingly affected (Fig 2B). These results could possibly explain the improvement observed in motor parameters and in animal thriving (Fig 1).

Pharmacokinetic analysis is instrumental for explaining the effects of 144DG11 *in situ* regardless of its innate capacity to modify polyglucosans in isolated cells, the reason being that timing of arrival, distribution, and stability in the tissue are key determinants of the *in situ* activity of any pharmacological agent. To determine the distribution and kinetic parameters of 144DG11 in different tissues, we treated Gbe^ys/ys^ mice with 250 mg/kg 144DG11 via subcutaneous injection, as in our efficacy experiments. Mice were then sacrificed 0, 30, 60, 90, and 210 min postadministration and serum as well as brain, kidney, hind limb skeletal muscle, heart, liver, and spleen tissues were collected, homogenized, and extracted, and their 144DG11 levels were analyzed by liquid chromatography tandem mass spectrometry (LC‐MS/MS). The results are shown in Fig 2C. The differential effects of 144DG11 on glycogen and polyglucosan content in the different tissues match its differential distribution and dwell time in each respective tissue. The highest extent of polyglucosan/glycogen reduction was observed in the liver matching the highest dwell time/persistence of 144DG11 observed in the organ (estimated half‐life of more than 3 h). The heart and brain demonstrate intermediate levels of 144DG11. However, those levels persist up until 60 min postinjection, which might account for the 144DG11‐mediated reduction in polyglucosan and glycogen content observed in these tissues. The muscle, on the other hand, demonstrated only negligible accumulation of 144DG11, in agreement with lack of effect of the compound on muscle glycogen and polyglucosan content. Based on the sampling times used, time to C_max_ was 30 min for all the tissues studied indicating similar rate of absorption to all these tissues. The highest C_max_ is observed in liver and kidney matching their well‐established rapid perfusion. Expectedly, the lowest C_max_ was observed in the skeletal quadriceps muscle, which is known to be a poorly perfused organ.

### 144DG11 enhances carbohydrate metabolism and improves metabolic panel *in vivo*

**Figure 3:**
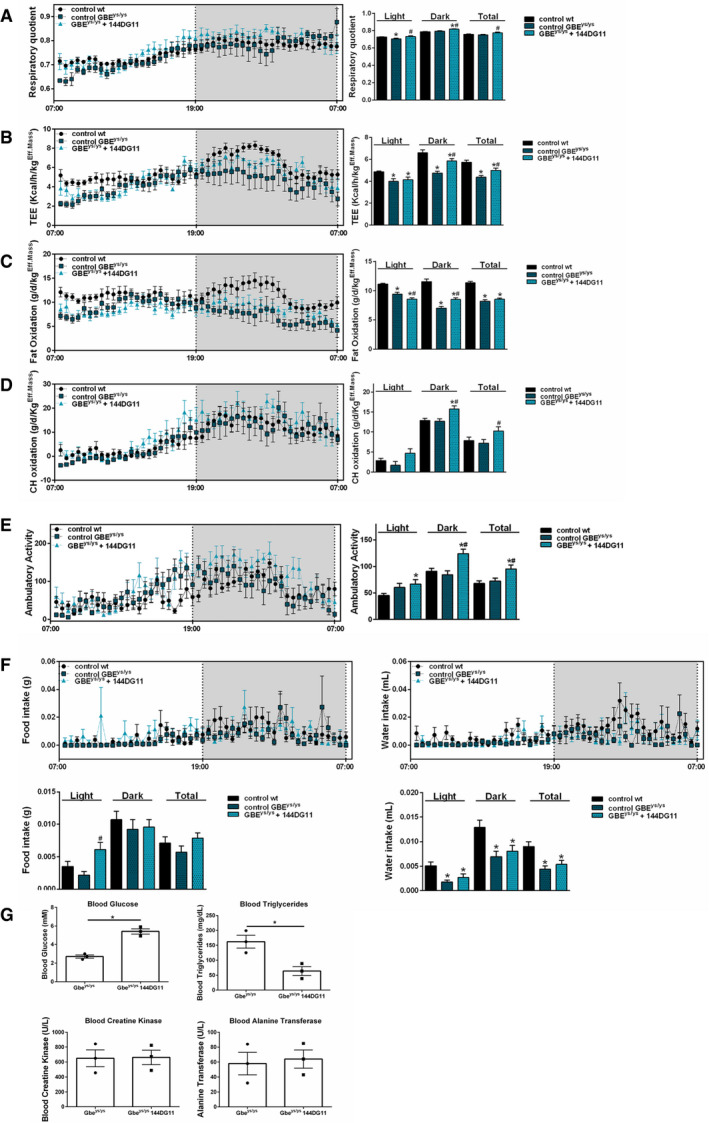
*In vivo* metabolic profile of mice treated with 144DG11 A–FMice were monitored over a 24‐hr period. Effective mass was calculated by ANCOVA (see Materials & Methods). Data are mean±SEM from nine‐month‐old mice (*n* = 11, wt vehicle‐treated, *n* = 6 Gbe^ys/ys^ vehicle‐treated, and *n* = 7 Gbe^ys/ys^ 144DG11‐treated). Vehicle‐treated Gbe^ys/ys^ mice demonstrate *lower* respiratory quotient (in the light) (A), total energy expenditure (TEE) (B), and fat oxidation (C) compared to wt controls. 144DG11 treatment increased these parameters (for fat oxidation only in the dark and total time). Carbohydrate oxidation and ambulatory activity, not significantly affected by the diseased state, were increased by 144DG11 even beyond wt control levels (D and E) (note, while 144DG11 increased carbohydrate oxidation in the light (D), *P* was only < 0.06). 144DG11 also reversed the decrease in meal size and water sip volume observed in Gbe^ys/ys^ mice as compared to wt control (F).GBlood metabolic panel based on *n* = 3, 9.5‐month‐old mice treated as indicated. Blood glucose was increased and blood triglycerides decreased in Gbe^ys/ys^ cells by 144DG11 (*P* < 0.05). Data information: *P < 0.05 *v* wt controls, ^#^ *P* < 0.05 *v* Gbe^ys/ys^ vehicle‐treated mice. Statistical differences were determined by two‐tailed t‐tests.

The effect of 144DG11 on various metabolic parameters was determined *in vivo* using indirect calorimetry. Fuel preference at the whole animal level is determined by the respiratory quotient (RQ, the ratio of CO_2_ produced to O_2_ consumed). Lower RQ indicates higher fat burn, while higher RQ indicates higher carbohydrate burn. As our results (Fig 3A) show, 144DG11 increased RQ to even higher levels than those of the wt animals. The parallel increases, induced by 144DG11, in total energy expenditure (Fig 3B) and carbohydrate burning at the expense of fat burning (Fig 3C and D) suggest that 144DG11 stimulates glycogen mobilization, which is a therapeutic advantage since Gbe^ys/ys^ mice store glycogen as insoluble and pathogenic polyglucosan. Stimulation of ambulatory activity (Fig 3E) and of meal size (Fig 3F) are in line with this observation of stimulation of carbohydrate catabolism in affected animals by 144DG11. Taken together, the increased fuel burning and food intake indicate that 144DG11 can improve metabolic efficiency in the affected animals.

We further tested whether 144DG11 is able to correct the hypoglycemia and hyperlipidemia observed in Gbe^ys/ys^ mice (Orhan Akman *et al*, 2015). Such an effect is expected from an agent capable of inducing the catabolism of liver glycogen with an ensuing rise in blood glucose. Our blood biochemistry test results of 9.5‐month‐old Gbe^ys/ys^ mice demonstrate that upon treatment with 144DG11, the characteristic hypoglycemia and hyperlipidemia of the mice were corrected to control levels (Fig 3G). Muscle (creatine kinase) and liver (alanine transferase) functions were not affected by this treatment (Fig 3G).

### 144DG11 enhances catabolism in glycogen overloaded APBD patient cells

**Figure 4:**
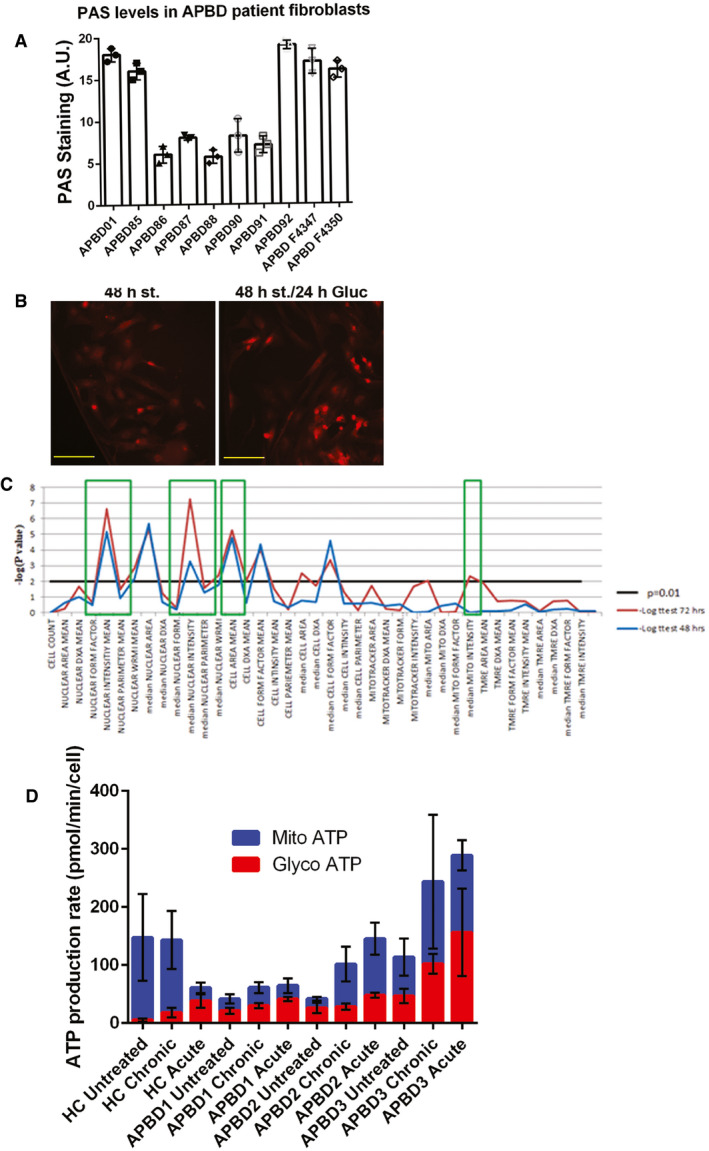
Glycogen burden in APBD fibroblasts and effect of 144DG11 on ATP production PAS staining for total glycogen in skin fibroblasts from different APBD patients. Staining fluorescence was quantified by InCell2200 (see Methods). Means (± SEM) are statistically different from each other (*P* < 0.0001, one‐way ANOVA). *n* = 3 technical replicates from each patient indicated at the x‐axis were used.PAS staining for total glycogen in APBD87 fibroblasts glucose starved for 48 h (*left*), or glucose starved and then replenished for the last 24 h to induce glycogen burden (*right*). Image acquisition performed by Nikon Eclipse Ti2 microscope using a 40x PlanFluor objective and CY3 filter. Scale bar, 100 µm.Image‐based multiparametric phenotyping (see Matrials & Methods) of APBD fibroblasts under 48 h glucose starvation (blue), or starvation and glucose replenishment as in (B) (red). Black line, level of significance *P* = 0.01. Green boxes indicate parameters whose extent of change was increased by glucose supplementation after starvation.Glycolytic (red) and mitochondrial (blue) ATP production determined by Agilent’s Seahorse machine and real‐time ATP rate assay kit. HC and APBD patient fibroblasts were serum/glucose‐starved for 48 h, and then, full medium was replenished for 24 h without (untreated) or with (chronic) 50 µM 144DG11. Acute, 50 µM 144DG11 was added on assay for 20 min after 24 h of serum/glucose replenishment. Readings were normalized to cell number as determined by crystal violet staining. Shown are mean and SEM values based on *n* = 3‐6 technical repeats. For all experiments (HC, *P* < 0.01; APBD1, *P* < 0.02; APBD2, *P* < 0.09; APBD3, *P* < 0.11; one‐way ANOVA with Sidak’s post hoc correction for multiple comparisons), glycolytic ATP production was increased, as compared to untreated control, by acute, but not chronic, supplementation of 144DG11. Mitochondrial ATP production was increased only by acute supplementation of 144DG11 to APBD2 fibroblasts (*P* < 0.06).

The RQ shift toward carbohydrate catabolism observed *in vivo* prompted us to investigate whether carbohydrate catabolism is also up‐modulated intracellularly. To that end, and especially since glycogen levels are highly variable among fibroblasts derived from different APBD patients (Fig 4A), we first aimed at inducing a physiological glycogen overload, or glycogen burden, condition, equivalent to the one found in tissues. We found that glycogen burden can be produced by 48 h glucose starvation followed by replenishment of the sugar for 24 h, which possibly induces accelerated glucose uptake with ensuing glycogen synthesis. This starvation/replenishment condition indeed increased intracellular glycogen levels, as demonstrated by PAS staining (Fig 4B). Furthermore, a multiparametric high‐content imaging‐based phenotyping analysis revealed that under glycogen burden conditions, cell area, nuclear intensity, and, importantly, mitochondrial mass features (see boxes in Fig 4C) deviated from healthy control (HC) more than glucose starved‐only cells did. Therefore, we selected this glycogen burden condition to analyze catabolism at a cell level using Agilent’s Seahorse ATP Rate Assay. Our results (Fig 4D) show that at the cell level, 144DG11 increased not only overall ATP production, but also the relative contribution of glycolytic ATP production at the expense of mitochondrial (OxPhos) ATP production. This phenomenon was observed in both HC and APBD patient skin fibroblasts. Acute on assay supplementation of 144DG11 was more effective at augmenting the glycolytic contribution to ATP production than 24 h pretreatment with the compound. These results suggest that glucose derived from the 144DG11‐mediated enhanced carbohydrate catabolism is exploitable for ATP production.

### 144DG11 binds to the lysosomal membrane protein LAMP1

**Figure 5:**
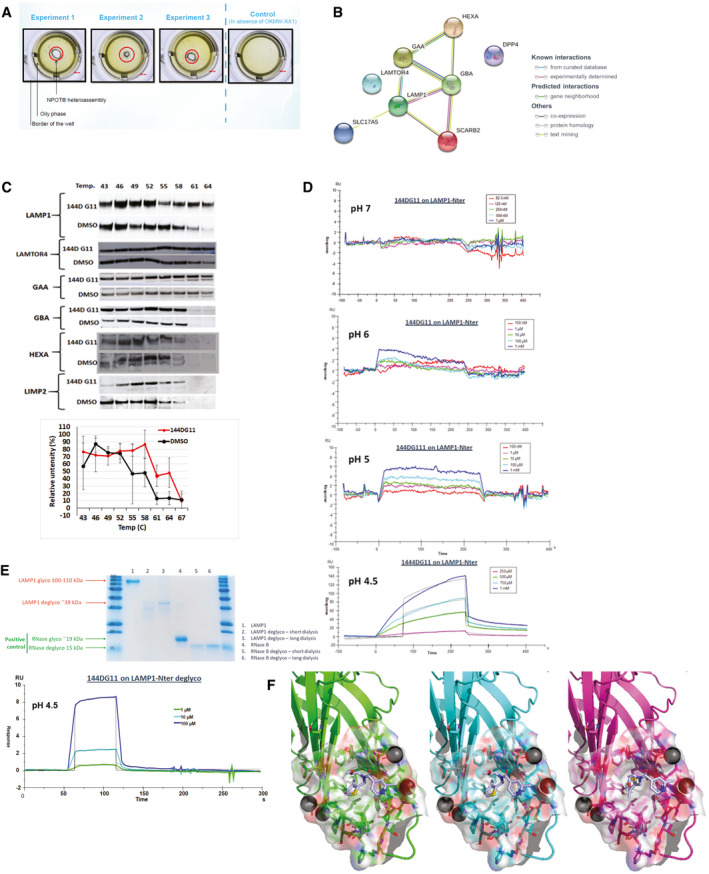
144DG11 interacts with LAMP1 and its associated interactome AHeteroassembly forms around 144DG11 and not around endogenous molecules as shown by the liquid crystals formed in experiments 1‐3 (see Materials & Methods).BSTRING network of targets at the interactome of 144DG11. Targets also show strong protein–protein interactions among themselves, as connectors show (see legend). SLC17A5, Solute Carrier Family 17 Member 5; LAMP1, Lysosomal Associated Membrane Protein 1; LAMTOR4, Late endosomal/lysosomal adaptor, MAPK and MTOR activator 4; GBA, Glucosylceramidase; SCARB2 (aka LIMP2), Scavenger Receptor Class B Member 2; DPP4, Dipeptidyl peptidase‐4; GAA, Lysosomal alpha‐glucosidase; HEXA, Beta‐Hexosaminidase Subunit Alpha.CCellular thermal shift assay (CETSA) of different targets of the 144DG11heteroassembly (B). Only LAMP1 was significantly protected by 144DG11 from heat‐mediated denaturation (see right shift in its Tm in the lower panel) suggesting its specific interaction with 144DG11. *n* = 3 biological replicates. Error bars represent S.D.D–FSurface plasmon resonance assays demonstrate dose and pH‐responsive interaction between LAMP1 and 144DG11. Full interaction (but with apparent K_D_ of 6.3mM) was demonstrated only at the lysosomal pH 4.5‐5. (E) *Upper panel*, LAMP1 was deglycoslated as detailed in Materials & Methods. RNase B is a positive control for a glycosylated protein. Glycosylation status is shown after short (24 h) or long (72 h) dialysis. The results demonstrate a full deglycosylation of both LAMP1 luminal part and RNase B, with unique bands appearing after a long dialysis. *Lower panel*, surface plasmon resonance, performed as in (D), showing that the luminal domain of deglycosylated LAMP1 specifically binds 144DG11 with apparent K_D_ of 52.5 nM. (F) Three binding modes of 144DG11 (gray) according to LAMP1 grids predicted by SiteMap (green), fPocket (cyan) and FtSite (magenta). Two out of three binding modes (SiteMap and fPocket) are identical. In FtSite part of the molecule went through a rotation relative to the other two. See Materials & Methodss5.

We next investigated the mechanism of action of 144DG11. To that end, we first decided to determine its molecular target. Nematic protein organization technique (NPOT, Inoviem, Ltd.) was applied to homogenates of APBD patient fibroblasts. The NPOT analysis revealed protein heteroassemblies uniquely generated around 144DG11 only when it was added to the cell homogenates (Fig 5A, Appendix Fig S2). The next step in this analysis identified the interactome of protein targets interacting with 144DG11 in APBD patients’ fibroblasts. Interestingly, as revealed by Inoviem’s gene ontology analysis based on several bioinformatic tools, proteins in the heteroassembly interacting with 144DG11 in APBD patient fibroblasts are autophagy or lysosomal proteins (Fig 5B). Moreover, we tested by cellular thermal shift assay (Jafari *et al*, 2014) the specific interaction of 144DG11 with 6 of the 8 targets discovered by NPOT. Our results (Fig 5C) suggest that LAMP1, and not other protein targets, directly interacts with 144DG11. This finding is related to a novel pathogenic hypothesis connecting cellular glycogen overload with glycogen trafficking to lysosomes via starch‐binding domain‐containing protein 1 (Sun *et al*, 2016). To validate 144DG11’s interaction with LAMP1, we used surface plasmon resonance (SPR) technology. Our SPR data (Fig 5D) show a specific and dose‐dependent binding of 144DG11 to the luminal portion of LAMP1 only at the lysosomal pH 4.5‐5 and not at the cytoplasmic pH 7, with some binding starting at the intermediate pH 6. Taken together, these results constitute a strong and acceptable evidence that the specific target of 144DG11 is the type 1 lysosomal protein LAMP1, widely used as a lysosomal marker and a known regulator of lysosomal function. However, the apparent K_D_ of this binding was relatively high (6.3 mM), which we ascribed to the slow k_on_ (rate of association in Fig 5D, pH 4.5). We hypothesized that this slow rate of association could be explained by inhibited diffusion of 144DG11 due to the bulky oligosaccharides at the glycosylation sites. Therefore, we repeated the SPR experiments with a chemically deglycosylated luminal LAMP1 domain. Indeed, deglycosylated LAMP1 bound 144DG11 with an apparent K_D_ of 52.5 nM, possibly due to profound structural changes induced by the deglycosylation (Fig 5E). We further investigated 144DG11 binding to LAMP1 by structure‐based computational docking. In the search for a putative binding site for 144DG11 in LAMP1, we analyzed the N‐ and C‐terminal subdomains of its luminal domain (residues A29‐R195 and S217‐D378, respectively), which have a similar topology (Terasawa *et al*, 2016). These domains were modeled (Materials & Methods) at the intralysosomal pH 5 based on the known crystal structure of mouse LAMP1 C‐terminal domain (PDB ID 5gv0). Possible binding sites were identified by three different computational tools: SiteMap, FtSite and fPocket, and docking computations of 144DG11 and a set of decoy molecules (Appendix Table S2) were performed to every putative binding site by the Glide algorithm. Fig 5F shows the 144DG11 LAMP1 putative binding pocket (residues F50‐D55, N62, L67, F118, Y120‐L122, T125, L127‐S133, N164‐V166), predicted by all three tools and with high selectivity to 144DG11 according to the docking results. Prediction of the same binding site by three different programs is very rare and thus strongly suggests that 144DG11 binds to the specified site at the N‐terminal of LAMP1. As can be seen in Fig 5F, Asn‐linked oligosaccharides face away from the predicted 144DG11 binding site and are therefore not expected to directly interfere with its binding. However, they might still affect 144DG11 diffusion in agreement with the significantly reduced K_D_ in deglycosylated LAMP1 (Fig 5E).

### 144DG11 enhances LAMP1 knockdown‐induced autolysosomal degradation and catabolism of glycogen

**Figure 6:**
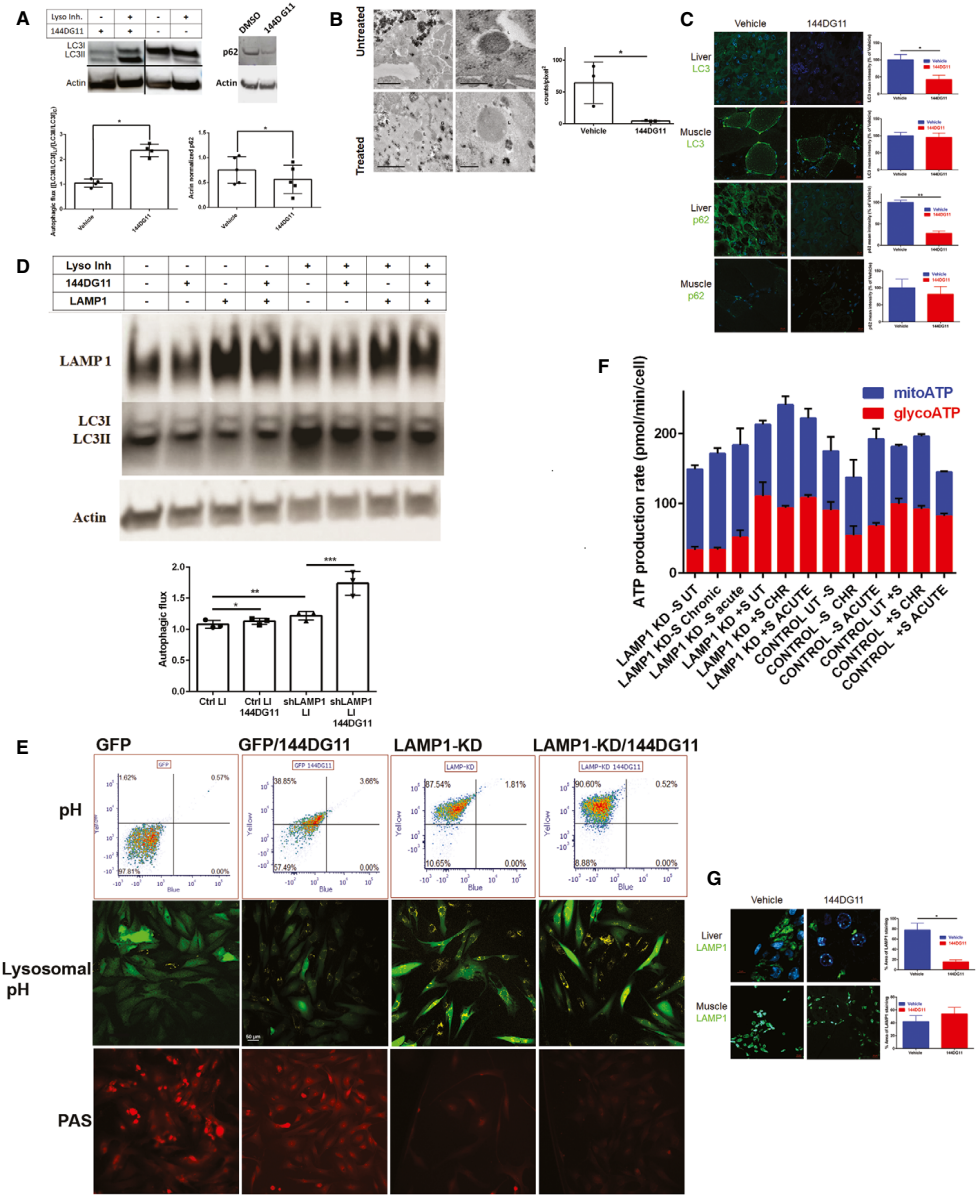
LAMP1‐KD and 144DG11 enhance autophagic flux *Left panel*, Autophagic flux, determined by the extent of lysosomal inhibitor‐dependent increase in the ratio of lipidated to non‐lipidated LC3 (LC3II/LC3I), is increased by 144DG11 (50µM, 24h). *Right panel*, 144DG11‐mediated increase in autophagic flux, demonstrated by enhanced degradation of the autophagy substrate p62. Shown are representative immunoblots (grouped from different gels) and densitometric quantifications ±s.d. (for LC3II/LC3I, *n* = 4 biological replicates, *P < 0.0023; for p62, *n* = 5 biological replicates, *P < 0.0099; two‐tailed t‐test).
*Left panel*, TEM images of liver tissue from vehicle or 144DG11‐treated Gbe^ys/ys^ mice. High‐magnification (*right*, scale bars=200 nm) and low‐magnification (*left*, scale bars=1 µm) images show higher levels of glycogen/polyglucosan in lysosomes and cytosol, respectively. *Right panel*, quantification (± s.d) of lysosomal glycogen particles (*n* = 3 biological replicates, *P < 0.03, two‐tailed *t*‐test). G, Glycogen/polyglucosan; L, Lysosomes; M, Mitochondria.144DG11 reduces LC3 and p62 in mouse liver, but not muscle (*n* = 3 biological replicates, *P < 0.04 for LC3, **P < 0.0007 for p62; two‐tailed t‐test, SEM). Scale bars, 10 µm.LAMP1 knockdown increases autophagic flux, which is further facilitated by 144DG11 (see densitometric quantification ±SEM (*n* = 3 biological replicates, *P < 0.1; **P < 0.05; ***P < 0.01 (two‐tailed t‐tests)). Immunoblots show LAMP1, LC3II/I, and actin in LAMP1 knocked down and control APBD fibroblasts treated or not with 144DG11 and lysosomal inhibitors (LI).LAMP1‐KD and 144DG11 treatment cause lysosomal acidification. *Upper panel*, flow cytometry results showing that 144DG11 slightly increased acidification (yellow to blue median fluorescence ratio (Y/B)) in control, GFP‐transduced, APBD fibroblasts (Y/B(GFP/144DG11)>Y/B(GFP), *P* < 0.12), but significantly acidified LAMP1‐KD, GFP‐shLAMP1‐transduced, APBD fibroblasts (Y/B(LAMP1‐KD/144DG11)>(Y/B(LAMP1‐KD), *P* < 0.03). LAMP1‐KD itself led to the most significant acidification (Y/B (LAMP1‐KD)>Y/B(GFP), *P* < 0.007). *n* = 3, two‐tailed t‐tests. *Middle panel* Lysosensor staining of the corresponding cells. Yellow fluorescence intensity correlates with acidification. *Lower panel*, PAS (glycogen) staining of the corresponding cells. Scale bars, 50 µm.ATP production rates (Fig 4B) ± SEM in serum‐starved (−S) and non‐starved (+S) LAMP1‐KD and GFP (Control) cells, untreated (UT), or treated for 24 h (CHR, chronic) or on assay (acute) with 144DG11. See text (*144DG11 enhances LAMP1 knockdown‐induced autolysosomal degradation and catabolism of glycogen*, third paragraph) for results of statistical analysis (multiple t‐tests, one‐way ANOVA with Sidak’s post hoc corrections, *n* = 3 biological replicates).144DG11 reduces lysosomal (LAMP1 positive) area in liver, but not muscle, of Gbe^ys/ys^ mice (*n* = 3 biological replicates, *P < 0.01, two‐tailed t‐test, SEM).Scale bars, 5 µm for liver (upper left panel), and 10 µm for muscle (lower left panel). Right panel shows quantification of the left panel. Source data are available online for this figure.

144DG11 increased autophagic flux in APBD primary fibroblasts. This is demonstrated by an increased sensitivity to lysosomal inhibitors in the presence of 144DG11. As can be seen in Fig 6A, lysosomal inhibitors increase the LC3ii/LC3i ratio (autophagic halt) more in 144DG11‐treated than in untreated cells. Increase in autophagic flux by 144DG11 was also illustrated by lowering the level of the autophagy substrate p62 (Fig 6A). Moreover, transmission electron microscope analysis of liver sections of the APBD modeling Gbe^ys/ys^ mice demonstrates a decrease in lysosomal glycogen following treatment with 144DG11 (Fig 6B). This result is consistent with the observed decrease in total LC3 and p62 levels in Gbe^ys/ys^ liver (Fig 6C), which demonstrates increased autophagic degradation (*e.g*., Zhang *et al* (2017)). On the other hand, LC3 and p62 levels in muscle tissue, unaffected by the poorly distributed 144DG11 (Fig 2A and C), were not influenced by 144DG11 (Fig 6C).

To determine the functional importance of the interaction between 144DG11 and LAMP1, we knocked down the latter using a lentiviral vector carrying GFP tagged shRNA against LAMP1. As LAMP1 knockdown (KD) becomes cytotoxic 24 h postexpression (or 96 h postlentiviral infection), LAMP1‐KD experiments in Fig 6C and D were conducted under 24 h serum starvation condition, without glucose replenishment (Fig 4), to both induce autophagy and maintain cell viability. We expected LAMP1‐KD to neutralize the effect of 144DG11 allegedly mediated by its interaction with LAMP1. Surprisingly, however, supplementation of 144DG11 to LAMP1 knocked down (*N.B*., not knocked out) cells enhanced the knockdown effect: Autophagic flux, enhanced by LAMP1‐KD, was further enhanced by the LAMP1 interacting 144DG11 (Fig 6D). The observation that 144DG11 enhances LAMP1‐KD effect suggests that the interaction of 144DG11 with LAMP1 is inhibitory, as many other small molecule–protein interactions are. Furthermore, to test whether LAMP1‐KD and 144DG11 enhanced autophagic flux by improving lysosomal function, we quantified lysosomal acidification using the pH ratiometric dye Lysosensor, which quantifies pH based on the yellow/blue emission ratio. Our results show that both LAMP1‐KD and 144DG11 treatment (in GFP and LAMP1‐KD APBD cells) led to lysosomal acidification, but more so LAMP1‐KD. As our confocal microscope is missing the 360 nm excitation line, we were unable to detect blue fluorescence microscopically. Therefore, we show by flow cytometry (Fig 6E, upper panel) the overall cellular acidification as an increase in 375 nm‐excited yellow/blue emission, and by confocal microscopy that this acidification is associated with brighter yellow fluorescence in lysosomes (Fig 6E, middle panel). Importantly, as demonstrated by PAS staining, LAMP1 knockdown also reduced cellular glycogen levels, an effect which was slightly enhanced by 144DG11 in APBD fibroblasts transduced with both GFP control and shLAMP1‐GFP lentiviruses (Fig 6E, lower panel).

To test the effect of LAMP1‐KD and 144DG11 on fuel utilization, we again used the ATP Rate Assay (Fig 4) in LAMP1‐KD and control APBD fibroblasts acutely or chronically treated with 144DG11. Our results (Fig 6F) show that starvation was more restrictive (lowered overall ATP production) in LAMP1‐KD (LAMP1‐KD‐S UT *v* LAMP1‐KD+S UT, *P* < 0.0001) than in GFP‐transduced controls (Control UT‐S *v* Control UT+S, *P* < 0.36). In LAMP1‐KD cells, starvation also increased the relative contribution of respiration to ATP production (78% in LAMP1‐KD‐S UT *v* 48% in LAMP1‐KD+S UT (orange bars)). These observations are in line with the higher ATP production efficiency of respiration as compared to glycolysis and with a possibly higher ATP demand of LAMP1‐KD, as compared to control cells, as suggested by their higher overall ATP production rate in basal conditions (*cf*. LAMP1‐KD+S UT with Control+S UT, *P* < 0.01). The effect of 144DG11 on LAMP1‐KD and control cells was in accordance with its selective increase of catabolic (ATP generating) autophagic flux in LAMP1‐KD cells, as compared to control cells (Fig 6D): In non‐starved conditions, supplementation of 144DG11 significantly increased total and respiratory ATP production in LAMP1‐KD cells (*cf*. LAMP1‐KD+S UT with LAMP1‐KD+S Chronic (*P* < 0.03 for total, *P* < 0.0008 for respiratory) and LAMP1‐D+S Acute (*P* < 0.01 for total and respiratory)), while it only slightly influenced ATP production, and even acutely decreased it, in control cells (*cf*. Control UT+S with Control+S Chronic (*P* < 0.1) and Control UT+S Acute (*P* < 0.0008 for decrease)). Under starved conditions, control cells only increased respiratory ATP production in response to the transient effects of acutely supplemented 144DG11 (*cf*. Control UT‐S with Control‐S Acute, *P* < 0.004). No significant effect of chronic supplementation of 144DG11 was observed in control cells (*cf*. Control UT‐S to Control‐S Chronic, *P* < 0.3). In contrast, starved LAMP1‐KD cells increased both respiratory and glycolytic ATP as a response to acute supplementation of 144DG11, possibly reflecting short‐term diversion of glucose derived from glycogen degradation to glycolysis (*cf*. LAMP1‐KD‐S UT with LAMP1‐KD‐S Acute (*P* < 0.0003 for glycoATP, *P* < 0.003 for mitoATP). In response to chronically administered 144DG11, only respiratory ATP production increased (*cf*. LAMP1‐KD‐S UT with LAMP1‐KD‐S Chronic (*P* < 0.15 for glycoATP, *P* < 0.0002 for mitoATP) in LAMP‐KD cells. In addition, LAMP1 staining area was reduced by 144DG11 only in liver sections (Fig 6G), possibly associated with the compound‐mediated improvement of lysosomal function.

### 144DG11 reduces lysosomes and restores aberrant mitochondrial features

**Figure 7:**
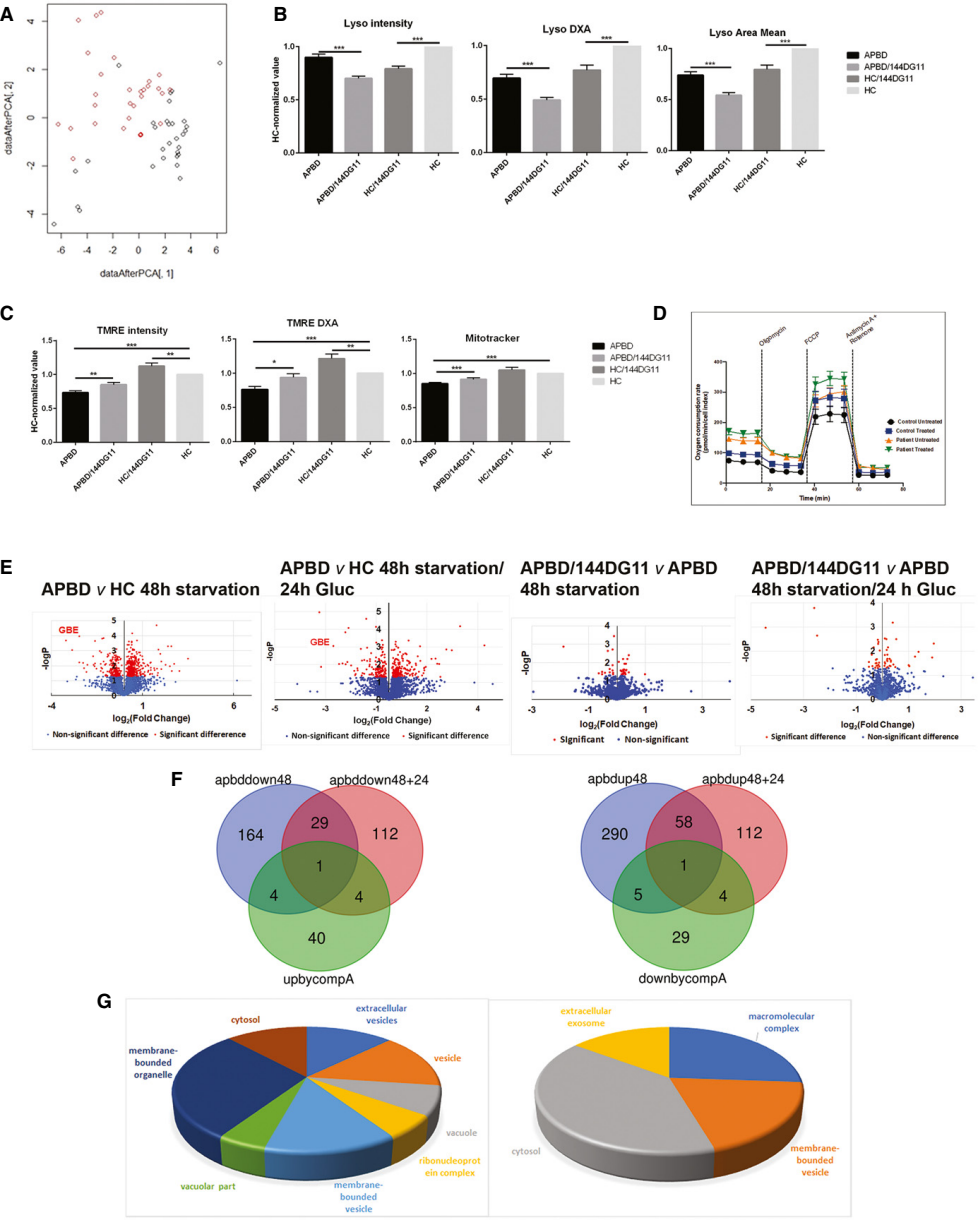
Phenotypic and proteomic effects of 144DG11 A, BPrincipal component analysis of IBP parameters in HC (black circles) and APBD (red circles) fibroblasts. (B) Morphological characterization of lysosomes based on lysotracker staining of *n* = 4 HC and *n* = 4 (biological replicates) APBD patient skin fibroblasts starved for 48 h and treated or not with 50 uM 144DG11 for 24 h. Values are deviations from untreated HC. 144DG11 has reduced lysotracker mean intensity, integrated intensity (DXA), and area in both HC and APBD fibroblasts (***P < 0.0001, one‐way ANOVA with Sidak’s multi‐comparison post hoc test).BFunctional (mitochondrial membrane potential (TMRE parameters)) and biomass (mitotracker) characterization of mitochondria in HC and APBD cells treated and analyzed as in (B). Mean (***P < 0.0001) and integrated (***P < 0.0006) TMRE intensities, and mitochondrial biomass (***P < 0.0001), were reduced in APBD *v* HC. 144DG11 increased TMRE mean (**P < 0.009, ** *P* < 0.006) and integrated (*P < 0.01, **P < 0.001) intensities in APBD and HC cells, respectively, and mitotracker intensity in APBD cells only (***P < 0.0001). Data represent *n* = 3 biological replicates. Statistical differences analyzed by one‐way ANOVA with multiple comparisons.CHC and APBD patient cells, starved for 48 h and then treated with 50 uM 144DG11 for 24 h in full medium, were subjected to bioenergetic characterization. 144DG11 treatment significantly increased basal and maximal (following FCCP) oxygen consumption rate (OCR), and coupling efficiency ((OCR drop following Oligomycin)/(Basal OCR)), in both HC and APBD fibroblasts, and ATP production (basal OCR – oligomycin OCR) only in APBD fibroblasts (Fig EV3). A representative experiment (*n* = 3 biological replicates) based on *n* = 6 technical replicates.DVolcano plots of the proteins affected by APBD and 144DG11 under starvation or glycogen burden conditions. Analysis is based on cells derived from *n* = 3 APBD and *n* = 3 HC subjects.EVenn diagrams of proteins down‐modulated by APBD and up‐modulated by 144DG11 and *vice versa* under starvation (48) and glycogen burden (48 + 24) conditions.FGene ontology of proteins up‐modulated (left) and down‐modulated (right) by 144DG11. Data information: All error bars represent SEM.

**Figure EV3:**
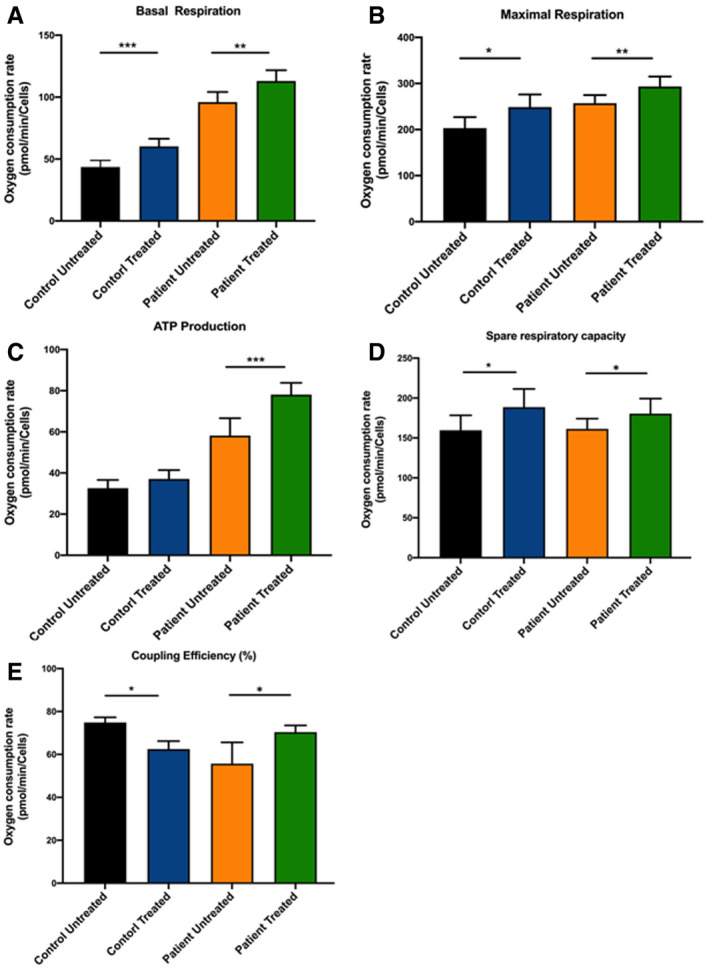
Bioenergetic parameters of 144DG11‐treated cells Basal respiration in the indicated groups calculated as the mean OCR from the initiation of the experiment until first injection of oligomycin. 144DG11 led to a significant increase in basal respiration in both healthy control (***P < 0.007) and APBD patient (**P < 0.02) cells.Maximal respiration, defined as the difference between OCR values after FCCP and rotenone/antimycin supplementations, is increased by 144DG11 in both HC (*P < 0.04) and APBD patient (**P < 0.03) cells.ATP production, defined as the difference in OCR values between basal and post oligomycin levels, is increased by 144DG11 only in APBD patient cells (***P < 0.006).Spare respiratory capacity, defined as the difference between maximal (post FCCP) OCR and basal OCR values, was slightly (not significantly) increased by 144DG11 in both HC (*P < 0.1) and APBD patient (*P < 0.13) cells.Coupling efficiency, defined as the quotient (OCR following Oligomycin)/(Basal OCR), was increased by 144DG11 in HC (*P < 0.01) and APBD patient (*P < 0.02) cells. Data information: All analyses are based on mean values of *n* = 3 biological replicates ± SEM. Statistical analysis was done by one‐way ANOVA with Dunnett’s post hoc tests.

As we showed that the mode of action of 144DG11 involves lysosomal catabolism which increases ATP production, we decided to investigate whether the cellular features modulated by 144DG11 are relevant to its catabolic effects. As a first step, we determined that APBD and HC fibroblasts are phenotypically different in general and that the APBD phenotype is significantly more heterogeneous (Fig 7A). This was done using image‐based high‐content analysis (HCA) of thousands of cells per sample in the InCell 2200 image analyzer, followed by principal component analysis. 45 cellular features from 17 APBD and 5 age matching HC fibroblasts were analyzed under environmental control conditions. Our findings suggest plasticity of the affected phenotype and thus predisposition to interventional restoration (*e.g*., by 144DG11). As 144DG11 targeted LAMP1 and lysosomal and catabolic functions (Figs 4, 5, 6), we focused on its effects on lysosomes and mitochondria, as analyzed by imaging and bioenergetic characterization. Fig 7B shows that 144DG11 reduces lysosomal staining intensity and area under starvation. This lysosomal reduction, observed also in healthy as compared to lysosomal impaired cells (de Araujo *et al*, 2020), might be associated with the compound’s mediated improvement of autophagic flux, induced in starvation, and lysosomal function (Fig 6). Mechanistically, lysosomal reduction by 144DG11 could be mediated by its effect on LAMP1, a major component of the lysosomal membrane which maintains its integrity. Fig 7C shows that 144DG11 has also hyperpolarized the mitochondrial membrane potential (MMP), depolarized by the diseased state in APBD, and increased mitochondrial biomass, also reduced by the diseased state. Both results are in accordance with possibly increased mitochondrial fueling by the enhanced autophagic catabolism. To test this possibility, we conducted a bioenergetic analysis of the effect of 144DG11 in glycogen burden conditions, demonstrated to enhance its effects (Fig 4). Our results (Figs 7D and EV3) show that 144DG11 has increased basal and maximal respiration, as well as mitochondrial ATP production, coupling efficiency and capacity to offset proton leak, or respiratory control ratio (state 3/state 4 respiration).

To investigate the global therapeutic impact of 144DG11, we analyzed the effect of the disease and the treatment on protein expression under different conditions (Dataset EV1). As shown in Fig 7E, under 48 h starvation, 12.2% and 6.8% of the 2,898 proteins analyzed were, respectively, up‐ and down‐modulated in APBD patient as compared to HC cells. Interestingly, endocytosis, a pathway implicated in lysosomal biogenesis and function, is a KEGG pathway up‐modulated in APBD cells (Appendix Fig S3), while oxidative phosphorylation is down‐modulated in APBD cells (Appendix Fig S4). As an important control, GBE was indeed down‐modulated in the APBD cells (Fig 7D). When starvation was followed by glucose supplementation (glycogen burden, Fig 4), only 6% of the proteins were up‐modulated and 5% down‐modulated, possibly suggesting a more specific subset of proteins was required for managing the excess glycogen burden. For instance, autophagy proteins (Fyco1, Rab12, Rab7A, PIP4K2B, SQSTM1, and SNAP29) were only up‐modulated in APBD cells following glycogen burden (Dataset EV1). We then investigated the proteomic effect of 144DG11 in starved (48 h starvation) and glycogen overladen (48 h starvation/24 h Gluc) APBD cells, which, respectively, modified only 1.7% and 1.3% of all proteins. The apparently corrective effect of 144DG11 can be uncovered by proteins down‐modulated or up‐modulated by the APBD diseased state, which were inversely up‐modulated (Table 1) or down‐modulated (Table 2) by 144DG11 (Fig 7F). The discovered proteins (49 up‐modulated, 39 down‐modulated, Fig 7F) were analyzed by the DAVID functional annotation tool according to the cellular component category, which included the highest number of proteins. Proteins up‐modulated by 144DG11 belonged to 8 significant gene ontology (GO) terms, which included lysosomal, secretory pathways, and oxidative phosphorylation proteins (Fig 7G, left panel and Table 1) in accordance with the cell features modulated by the compound (Fig 7B and C). Interestingly, proteins down‐modulated by APBD and up‐modulated (“corrected”) by 144DG11 (light gray, Table 1) were the lysosomal glycosylation enzymes iduronidase and phosphomannomutase2 under glycogen burden, whereas under starvation those were the nucleic acid binding proteins GRSF1 and HNRPCL1, apparently not directly associated with glycogen and lysosomal catabolism (light gray, Table 1). The lipogenetic protein HSD17B12 was decreased by APBD and induced by 144DG11 under both conditions (dark gray, Table 1). Proteins down‐modulated by 144DG11 belonged to 4 GO terms, which included secretory pathways and macromolecular complexes (Fig 7G, right panel and Table 2). Proteins increased by APBD and contrarily reduced by 144DG11 belonged to lysosomal sorting (VPS16) and carbohydrate biosynthesis (NANS) in starved cells and to transcription (RUBL1), signal transduction (STAM2), and pH regulation (SLC9A1) in glycogen overladen cells. Interestingly, pharmacological inhibition of the Na^+^/H^+^ antiporter SLC9A1 induces autophagic flux in cardiomyocytes (Riaz *et al*, 2020) as does its down‐modulation in APBD fibroblasts by 144DG11 (Table 2). The protein down‐modulated by 144DG11 in both starved and glycogen burden conditions is the retrograde traffic regulator VPS51 (Table 2, dark gray) also implicated in lysosomal sorting. In summary, the APBD correcting effects of 144DG11 are at least partially related to lysosomal function whose modulation by the compound is well characterized here (Figs 5 and 6).

**Table 1:** *Homo sapiens* proteins up‐modulated by 144DG11

Uniprot Accession #	Protein Name	Extracellular vesicles^a^	Vesicle^d^	Vacuole^g^	Ribonucleoprotein complex^d^	Membrane‐bounded vesicle^d^	Vacuolar part^g^	Membrane‐bounded organelle^g^	Cytosol^h^
P84243	H3 histone family member 3A(H3F3A)	+	+			+		+	
Q9UK41	VPS28, ESCRT‐I subunit(VPS28)	+	+	+		+	+	+	+
P78537	Biogenesis of lysosomal organelles complex 1 subunit 1(BLOC1S1)	+	+	+		+	+	+	+
P10606	Cytochrome c oxidase subunit 5B(COX5B)	+	+			+		+	
Q9UBQ5	Eukaryotic translation initiation factor 3 subunit K(EIF3K)	+	+			+		+	+
Q9H0X4	Family with sequence similarity 234 member A(FAM234A)	+	+			+		+	
P15586	Glucosamine (N‐acetyl)‐6‐sulfatase(GNS)	+	+	+		+	+	+	
Q53GQ0^i^	Hydroxysteroid 17‐beta dehydrogenase 12(HSD17B12)	+							
P35475^j^	Iduronidase, alpha‐L‐(IDUA)	+	+	+		+	+	+	
Q2TAA2	Isoamyl acetate‐hydrolyzing esterase 1 homolog(IAH1)	+	+			+		+	
P38571	Lipase A, lysosomal acid type(LIPA)	+	+	+		+		+	
P11279	Lysosomal associated membrane protein 1(LAMP1)	+	+	+		+	+	+	+
O15305^j^	Phosphomannomutase 2(PMM2)	+	+			+		+	+
Q9UIW2	Plexin A1(PLXNA1)	+	+			+		+	
Q9UMY4	Sorting nexin 12(SNX12)	+	+	+		+		+	
P50502	Suppression of tumorigenicity 13 (colon carcinoma) (Hsp70 interacting protein)(ST13)	+	+			+		+	+
Q9NZD8	Spastic paraplegia 21 (autosomal recessive, Mast syndrome)(SPG21)		+	+		+	+	+	+
Q9UMX0	Ubiquilin 1(UBQLN1)		+	+				+	
Q12849^j^	G‐rich RNA sequence binding factor 1(GRSF1)				+			+	
O43583	Density regulated re‐initiation and release factor(DENR)				+			+	
Q9UBQ5	Eukaryotic translation initiation factor 3 subunit K(EIF3K)				+			+	
O60812^j^	Heterogeneous nuclear ribonucleoprotein C‐like 1(HNRNPCL1)				+			+	
P55795	Heterogeneous nuclear ribonucleoprotein H2 (H')(HNRNPH2)				+			+	
P17980	Proteasome 26S subunit, ATPase 3(PSMC3)				+			+	+
P62847	Ribosomal protein S24(RPS24)				+			+	+
Q9Y276	BCS1 homolog, ubiquinol‐cytochrome c reductase complex chaperone(BCS1L)							+	
Q86XP3	DEAD‐box helicase 42(DDX42)							+	
Q8NC56	LEM domain containing 2(LEMD2)							+	
Q14498	RNA binding motif protein 39(RBM39)							+	
P30876	RNA polymerase II subunit B(POLR2B)							+	
O95757	Heat shock protein family A (Hsp70) member 4 like(HSPA4L)							+	+
Q53GQ0	Hydroxysteroid 17‐beta dehydrogenase 12(HSD17B12)							+	
Q00978	Interferon regulatory factor 9(IRF9)							+	+
Q9NP80	Patatin like phospholipase domain containing 8(PNPLA8)							+	
O60331	Phosphatidylinositol‐4‐phosphate 5‐kinase type 1 gamma(PIP5K1C)							+	+
Q8IY67	Ribonucleoprotein, PTB binding 1(RAVER1)							+	
O43314	Diphosphoinositol pentakisphosphate kinase 2(PPIP5K2)								+
Q9BY32	Inosine triphosphatase(ITPA)								+

^b,e^Small, spherical organelles enclosed by a membrane.

^c,f^In mammalian cells equivalent to lysosomes. Vacuole is a more general term encompassing the yeast degradative organelle.

^a^ Vesicles in the extracellular region, such as exosomes originating from fusion of the limiting endosomal membrane of a multivesicular body with the plasma membrane.

^d^ Protein–RNA complexes such as those found in ribosomes and telomeres.

^g^ Distinct cellular structure with a defined function, bounded by a single or double membrane. Examples: nucleus, mitochondria, vacuoles, autophagosomes, and vesicles. Excludes the plasma membrane.

^h^ Cytoplasm without organelles, which contains particulate matter, such as protein complexes.

^i^ Protein up‐modulated by 144DG11 and down‐modulated by the diseased state (APBD) both under 48 h starvation and 48 h starvation followed by 24 h glucose replenishment.

^j^ Proteins down‐modulated by APBD, either after 48 h starvation, or after 48 h starvation and 24 h glucose replenishment, and up‐modulated by 144DG11.

**Table 2:** *Homo sapiens* proteins down‐modulated by 144DG11

Uniprot Accession #	Protein Name	Macromolecular complex^a^	Membrane‐bounded vesicle^b^	Intracellular part^c^	Extracellular exosome^d^
P51116	FMR1 autosomal homolog 2(FXR2)	+	+	+	+
Q9UBI6	G protein subunit gamma 12(GNG12)	+	+	+	+
Q9UNZ2	NSFL1 cofactor(NSFL1C)	+		+	
Q9Y265^e^	RuvB like AAA ATPase 1(RUVBL1)	+	+	+	+
Q9H269^e^	VPS16, CORVET/HOPS core subunit(VPS16)	+	+	+	
Q9UID3^f^	VPS51, GARP complex subunit(VPS51)	+	+	+	
Q9P2K1	Coiled‐coil and C2 domain containing 2A(CC2D2A)	+		+	
Q14746	Component of oligomeric Golgi complex 2(COG2)	+		+	
Q14008	Cytoskeleton associated protein 5(CKAP5)	+		+	
Q9Y295	Developmentally regulated GTP binding protein 1(DRG1)	+		+	
O95834	Echinoderm microtubule associated protein like 2(EML2)	+		+	
O95249	Golgi SNAP receptor complex member 1(GOSR1)	+	+	+	
P16402	Histone cluster 1 H1 family member d(HIST1H1D)	+		+	
Q9BW83	Intraflagellar transport 27(IFT27)	+		+	
P37198	Nucleoporin 62(NUP62)	+		+	
Q15365	Poly(rC) binding protein 1(PCBP1)	+	+	+	
Q9UBS8	Ring finger protein 14(RNF14)	+		+	
O75886^e^	Signal transducing adaptor molecule 2(STAM2)	+		+	
P19634^e^	Solute carrier family 9 member A1(SLC9A1)	+	+	+	
Q9NUQ6	Spermatogenesis associated serine rich 2 like(SPATS2L)	+		+	
Q15819	Ubiquitin conjugating enzyme E2 V2(UBE2V2)	+	+	+	
P46939	Utrophin(UTRN)	+	+	+	
Q9BRG1	Vacuolar protein sorting 25 homolog(VPS25)	+	+	+	
Q9Y5S2	CDC42 binding protein kinase beta(CDC42BPB)		+	+	+
Q9NR45	N‐acetylneuraminate synthase(NANS)		+	+	+
P61018	RAB4B, member RAS oncogene family(RAB4B)		+	+	
P52306	Rap1 GTPase‐GDP dissociation stimulator 1(RAP1GDS1)		+	+	+
Q04760	Glyoxalase I(GLO1)		+	+	+
Q6EMK4	Vasorin(VASN)		+	+	
Q06136	3‐ketodihydrosphingosine reductase(KDSR)			+	
Q14919	DR1 associated protein 1(DRAP1)			+	
Q9UBB4	Ataxin 10(ATXN10)			+	
Q96A33	Coiled‐coil domain containing 47(CCDC47)			+	
Q9NWY4	Histone PARylation factor 1(HPF1)			+	
Q9UI26	Importin 11(IPO11)			+	

^a^ A functional assembly which includes at least one protein and other macromolecules and/or ligands.

^b^ Small, spherical organelles enclosed by a membrane.

^c^ Cytoplasm without organelles, which contains particulate matter, such as protein complexes.

^d^ Internal vesicles of multivesicular bodies released into the extracellular milieu by fusion of the endosomal membrane with the plasma membrane.

^e^ Proteins up‐modulated by APBD, either after 48 h starvation, or after 48 h starvation and 24 h glucose replenishment, and down‐modulated by 144DG11.

^f^ Protein down‐modulated by 144DG11 and up‐modulated by the diseased state (APBD) both under 48 h starvation and 48 h starvation followed by 24 h glucose replenishment.

## Discussion

**Figure EV4:**
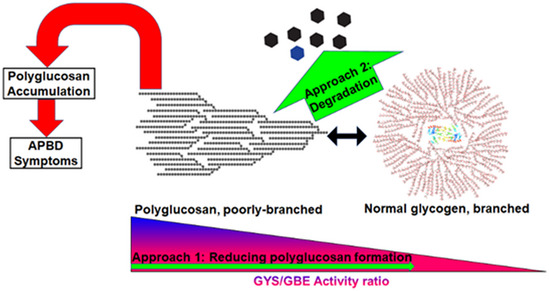
Therapeutic strategies for APBD Therapeutic approaches for APBD are based on reduction of the GYS/GBE activity ratio or on direct PG and glycogen degradation. Blue hexagon, glucose; black hexagon, glucose‐1‐phosphate.

This work shows that the HTS‐discovered hit 144DG11 can remedy APBD in *in vivo* and *ex vivo* models. Following 144DG11 treatment, we observed improvements in motor, survival, and histological parameters (Figs 1 and 2). As APBD is caused by an indigestible carbohydrate, these improvements suggested that 144DG11 affected carbohydrate utilization and thus encouraged us to conduct *in vivo* metabolic studies (Fig 3). To our knowledge, this is the first *in vivo* metabolic study in a GSD animal model. Since APBD mice store glycogen as insoluble polyglucosan, we tested by indirect calorimetry whether 144DG11 can influence the capacity of these animals to use alternative fuels (fat) instead of mobilizing glycogen. However, the increased RQ induced by 144DG11 suggested that instead of using fat, treated animals actually increased carbohydrate burn or that 144DG11 increased carbohydrate catabolism. This conclusion was supported by the 144DG11‐induced increases in total energy expenditure, ambulatory activity, and meal size—all in line with catabolic stimulation. Since Gbe^ys/ys^ mice and APBD patients store glycogen as insoluble and pathogenic polyglucosan, its catabolism constitutes a therapeutic advantage. Glycogen catabolism is also a preferred therapeutic strategy for the following reason: In theory, therapeutic approaches to APBD should target either PG formation, or degradation of preformed PG or glycogen (Fig EV4, approaches 1 and 2, respectively). PG formation depends on the balance between GYS and GBE activity—the higher the GYS/GBE activity ratio, the more elongated and less branched soluble glycogen would form, which would preferentially form PG, as compared to shorter chains (Sullivan *et al*, 2019). Degradation of pre‐existing PG and glycogen (PG precursor), on the other hand, as done by 144DG11, is a more direct target and is expected to be more efficacious than inhibition of de novo PG formation, as done by the GYS inhibitor guaiacol (Kakhlon *et al*, 2018), which spares pre‐made detrimental PG. Indeed, in a study in LD‐modeling mice, it was shown that conditional GYS knockdown after disease onset is unable to clear pre‐existing and detrimental Lafora PG bodies (Nitschke *et al*, 2021).

A key challenge in drug discovery is the determination of relevant targets and mechanism of action of drug candidates. To that end, we have applied here Inoviem’s NPOT protein target identification approach. This technique, recognized as a leading tool for identifying protein targets of small molecules (Comess *et al*, 2018), and which identified several therapeutically relevant targets (Beyrath *et al*, 2018; Walf‐Vorderwülbecke *et al*, 2018; Wang *et al*, 2018), identifies compound‐target interactions within the natural physiological environment of cells. This means that the entity identified is not the target *per se*, as in other technologies, but the primary target with its signaling pathway, or functional quaternary network. Determination of the cellular pathway modulated by the test compound, as done for 144DG11, is important for formulation of other drugs to the same pathway, which can significantly upgrade therapeutic efficacy in due course in the clinic. Moreover, NPOT can also confirm the specificity of target binding by filtering out promiscuous binders, and excluding binding to negative controls (in our case, negative compounds in the HTS) and to endogenous ligands (Fig 5A). Nevertheless, while by these criteria 144DG11 binding to LAMP1, and through it to its functional quaternary network (Fig 5B), was specific and manifested dose response and lysosomal pH dependence in the SPR validation (Fig 5D), its apparent LAMP1 binding K_D_ was relatively high (6.3 mM), which seemingly could be an impediment toward its clinical application. This issue can be coped with as follows: 1. The pharmacologically relevant finding is that 144DG11 specifically interacted with a lysosomal–autophagosomal interactome (Fig 5B) and that it was not toxic (Fig EV2, Appendix Table S1). This finding rules out non‐specific interaction with putative off‐targets, which is the main concern in low‐affinity (high K_D_) ligands, especially since this low affinity is probably a by‐product of oligosaccharide steric hindrance (Fig 5E). 2. A conventional approach to improve the affinity of low‐affinity pharmaceutical candidates is based on medicinal chemistry. In GSDs, such an approach was used for increasing the affinity of GYS inhibitors (Tang *et al*, 2020). However, as opposed to GYSs, whose reduction is relatively tolerable (Orho *et al*, 1998; Kollberg *et al*, 2007), the LAMPs belong to the house keeping autolysosomal machinery (Fig 5B), whose inhibition can compromise perinatal viability, as does, for instance, LAMP1‐KD without a compensatory rise in LAMP2 (Eskelinen *et al*, 2004; Eskelinen, 2006). Therefore, a high affinity LAMP1 inhibitor might be toxic, as was LAMP1‐KD to APBD fibroblasts (Fig 6), and the low affinity of the LAMP1 inhibitor we discovered, 144DG11, may actually constitute a clinical advantage by mitigating the repression of a household function. 3. The discovery of the LAMP1 containing heteroassembly (Fig 5B) as a functional network, rather than a single protein target, opens a therapeutic modality based on autophagy modulation, which actually expands the therapeutic target landscape. Autolysosomal network was discovered not only in Fig 5B, but also by image high‐content analysis, in conjunction with bioenergetic parameters, possibly modified by autophagy‐associated changes in fuel availability (Fig 7B and C). Other supports for the relevance of this pathway as a 144DG11 target come from our proteomics data (Fig 7D–G) and from the actual boost of autophagic flux by 144DG11 in cells (Fig 6).

**Figure EV5:**
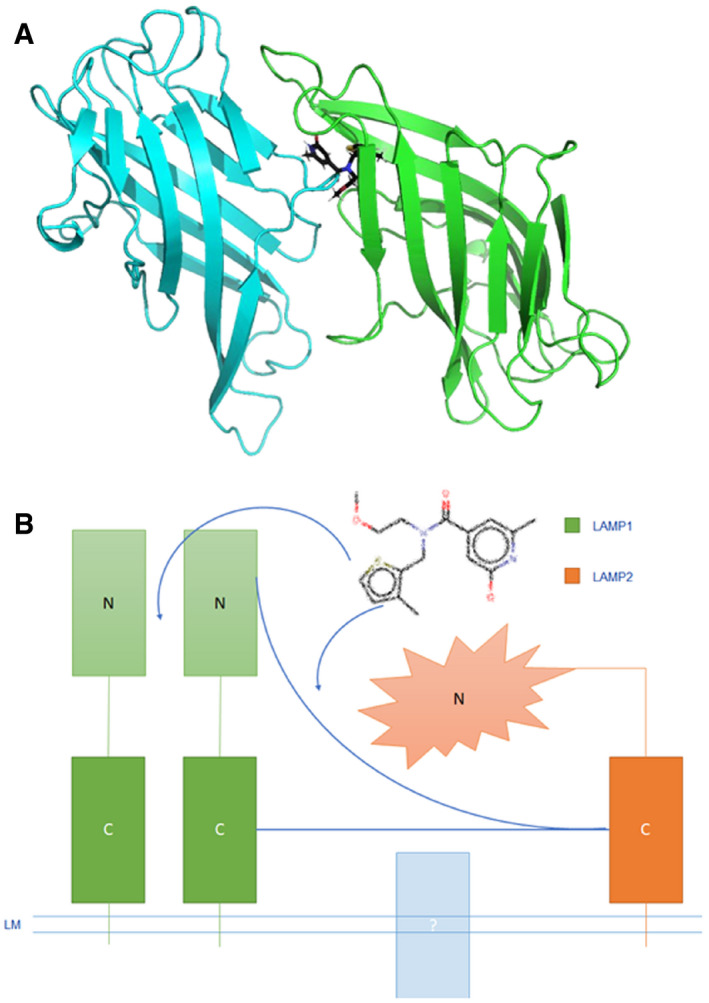
144DG11 at the LAMP1:LAMP1 interface To evaluate the probability that 144DG11 can interfere with LAMP1:LAMP1 interactions by binding to the predicted binding site at the LAMP1 N‐terminus, we performed LAMP1 N‐terminus:LAMP1 N‐terminus protein:protein docking computations (A). According to the three highest ranked solutions (the top ranked result is shown in (A)), 144DG11 putative binding site is located in the LAMP1:LAMP1 interface. The possibility that 144DG11 also inhibits LAMP1:LAMP2 interactions requires additional computations.
Predicted binding site for 144DG11 in LAMP1’s N‐terminal domain. Top ranked solution obtained by PATCHDOCK and FireDock servers. 144DG11, represented by black sticks, was docked to the putative binding site. LAMP1 N‐terminal chains are represented in green and cyan.Schematic of the lysosomal membrane (LM), LAMP1, LAMP2, and the potential inhibitor 144DG11, a possible ancillary membrane protein mediating LAMP1 interaction.

Mechanistically, LAMP1 is a type I lysosomal membrane protein which, together with LAMP2, plays a pivotal role in lysosome integrity and function (Eskelinen *et al*, 2004; Eskelinen, 2006). Consequently, LAMP1, but more so LAMP2 (González‐Polo *et al*, 2005), is also important for lysosomal involvement in the autophagy process. Therefore, LAMP1 knockdown is often associated with decreased autophagy (Krzewski *et al*, 2013; Polito *et al*, 2014). However, in agreement with our results, other works show that LAMP1‐KD actually increased autophagic function (González‐Polo *et al*, 2005; Schulze‐Luehrmann *et al*, 2016), which was also shown for another transmembrane lysosomal protein TMEM192 (Liu *et al*, 2012). These apparent disparities probably depend on cell type, assay conditions, and even the definition of autophagy, as autophagic flux is not always defined by susceptibility to lysosomal inhibitors (Klionsky *et al*, 2016). To predict the molecular mechanism of action of 144DG11 on LAMP1, we used structural‐computational techniques. Our computational results predict that the 144DG11 binding site is located at the LAMP1:LAMP1 interaction interface (Fig EV5A) (located at the N‐terminal domain) and suggest that the compound inhibits inter‐LAMP1 interaction. According to experimental data (Terasawa *et al*, 2016), truncation of the N‐terminal domain of LAMP1 impairs LAMP1/LAMP1 and LAMP1/LAMP2 assembly, while truncation of the more mobile LAMP2 N‐terminal domain leads to the opposite effect (Fig EV5B). Therefore, we may assume that LAMP1 N‐terminal domain promotes LAMP1/LAMP1 and LAMP1/LAMP2 interactions and that inhibition of LAMP1/LAMP1 or LAMP1/LAMP2 interactions at the N‐terminal domain, by 144DG11, would lower LAMP1 effective lysosomal membrane density. Thus, 144DG11 treatment can be hypothesized to be equivalent to LAMP1‐KD, which might explain its enhancement of the LAMP1‐KD effect. The slight increase (1.2 fold) in LAMP1 levels induced by 144DG11 (Table 1, Dataset EV1) probably reflects binding‐mediated stabilization (Fig 5C) and presumably does not significantly counteract 144DG11‐mediated reduction in membrane density, suggesting that it increases glycophagy by the documented (Eskelinen *et al*, 2004; Eskelinen, 2006) increase in LAMP2 in lysosomal membranes upon LAMP1‐KD. LAMP2 was observed to enhance autophagosome–lysosome fusion (and thus autophagic flux) by interaction with the autophagosomal peripheral protein GORASP2 (Zhang *et al*, 2019). Alternatively, spacing of the lysosomal membrane by LAMP1‐KD/144DG11 may enable glycogen import to the lysosome (and consequent degradation) by the STBD1 protein (Sun *et al*, 2016). Importantly, lysosomal glycogen degradation takes place in parallel with its cytoplasmic degradation (Adeva‐Andany *et al*, 2016) and, specifically, in a GSDIV mouse model, which also models APBD in mice, treatment with the recombinant human lysosomal enzyme acid α‐glucosidase alleviated disease in the liver (Yi *et al*, 2016).

In summary, this work demonstrates 144DG11 as a novel catabolic compound capable of degrading PG and over‐accumulated glycogen by activating the autophagic pathway. This study lays the groundwork for clinical use of 144DG11 in treating APBD patients who currently have no therapeutic alternative. Moreover, it positions 144DG11 as a lead compound for treating other GSDs through safe reduction of glycogen surplus.

## Materials and Methods

### *In* *vivo* experiments

We tested 144DG11 for its capacity to correct disease phenotypes in Gbe^ys/ys^ mice. Two arms, 5% DMSO vehicle and 144DG11, of initially *n* = 7‐9 animals each were used. These numbers were demonstrated retrospectively to provide sufficient power because, based on the means and SD obtained, a power of 80% is already attained at *n* = 5 animals/arm (Charan & Kantharia, 2013). Additional open field, gait, and extension reflex tests (Fig 1E and F, and H) also included a C57BL/6 wt control arm of *n* = 9 animals. Animals were excluded from the experiment if weight was reduced by > 10% between sequential weightings or by > 20% from initiation. Sample size was slightly reduced over time due to death. 150 µl of 250 mg/kg 144DG11 in 5% DMSO was injected twice a week. Injection was intravenous (IV) for the first month, followed by subcutaneous (SC) injection due to lack of injection space and scarring in animal tails. We initiated the injections either at the age of 4 months, two months prior to disease onset, assuming a preferred prophylactic effect, or at the 6 months age of onset for comparison (only Fig 1L–N). Treatment was continued until the age of 10 months. The effect of 144DG11 on the following motor parameters was tested approximately every 2 weeks: average duration in movement in an open field, extension reflex (the degree to which the hind paws open after holding the animal from the tail), and gait (stride length). At the end of these experiments, some of the mice were sacrificed, without prior fasting, by cervical dislocation and tissues from *n* = 3 wt, *n* = 7 Gbe^ys/ys^ vehicle‐treated, and *n* = 9 144DG11‐treated mice were collected, sectioned, fixed, and stained for diastase‐resistant PG by PAS (Kakhlon *et al*, 2018). Tissue glycogen was determined biochemically as described (Kakhlon *et al*, 2018). In addition, 144DG11 pharmacokinetic profile was determined by LC‐MS/MS in serum and tissues derived from *n* = 3 mice/time point (Materials & Methods). Experimenters were blinded to treatment allocation.

### Histological PG and glycogen determination

Brain, heart, quadriceps muscle, nerve fascicles (peripheral nerves), and liver tissues from wt and 144DG11 and vehicle‐treated Gbe^ys/ys^ animals were separated to characterize the histopathological effects of 144DG11. Tissues were extracted, fixed, embedded in paraffin, and sectioned. After deparaffinization, sections were treated for 5 min with 0.5% diastase to digest non‐polyglucosan glycogen, leaving behind polyglucosan. Sections were then washed, stained for polyglucosan with PAS and counterstained with hematoxylin, and analyzed by light microscopy, all as described in (Kakhlon *et al*, 2018). For biochemical glycogen determination, 100 mg of each tissue was subjected to alkaline hydrolysis and boiling followed by ethanol precipitation of glycogen. Glycogen was then enzymatically digested to glucose by amyloglucosidase (Sigma). Following digestion, total glycogen was determined based on the glucose content using the Sigma GAGO20 kit.

### Pharmacokinetics

For pharmacokinetic analysis, 100 μl serum as well as brain, kidney, hind limb quad muscle, heart, liver, and spleen tissues were collected, homogenized, and extracted with acetonitrile following established guidelines (Kapetanovic *et al*, 2006). Calibration curves were made with 0, 1, 10, 100, and 1,000 ng/ml 144DG11 in 1 mg/ml solutions of 4‐tert‐butyl‐2‐(4H‐1,2,4‐triazol‐4‐yl)phenol (ChemBridge) as internal standard (IS). Tissue samples were then dissolved in 1 mg/ml IS solutions and spiked with 0–1,000 ng/ml 144DG11 to generate standard curves from which tissue levels of 144DG11 were determined. Samples were analyzed by the LC‐MS/MS Sciex Triple Quad TM 5500 mass spectrometer.

### Multi‐parameter metabolic assessment

Metabolic and activity profiles of the mice were assessed by using the Promethion High‐Definition Behavioral Phenotyping System (Sable Instruments, Inc., Las Vegas, NV, USA) as described previously (Knani *et al*, 2016). Briefly, mice with free access to food and water were subjected to a standard 12‐h light/12‐h dark cycle, which consisted of a 48‐h acclimation period followed by 24 h of sampling. Respiratory gases were measured by using the GA‐3 gas analyzer (Sable Systems, Inc., Las Vegas, NV, USA) using a pull‐mode, negative‐pressure system. Air flow was measured and controlled by FR‐8 (Sable Systems, Inc., Las Vegas, NV, USA), with a set flow rate of 2,000 ml/min. Water vapor was continuously measured, and its dilution effect on O_2_ and CO_2_ was mathematically compensated. Effective body mass was calculated by ANCOVA analysis as described previously (Tschöp *et al*, 2011). Respiratory quotient (RQ) was calculated as the ratio of VCO_2_/VO_2_, and total energy expenditure (TEE) was calculated as *VO_2_ x (3.815 + 1.232 x RQ)*, normalized to effective body mass, and expressed as kcal/h/kg^Eff. Mass^. Fat oxidation (FO) and carbohydrate oxidation (CHO) were calculated as *FO = 1.69 x VO_2_ – 1.69x VCO_2_* and *CHO = 4.57 x VCO_2_– 3.23 x VO_2_* and expressed as g/d/kg^Eff. Mass^. Activity and position were monitored simultaneously with the collection of the calorimetry data using XYZ beam arrays with a beam spacing of 0.25 cm. Food and water intakes were measured, while calorimetric data were sampled.

### Imaging and image‐based phenotyping

APBD skin fibroblasts were seeded at 1,000 cells/well and cultured in specialized microscopy‐grade 96‐well plates (Grenier Bio‐One, Germany). Following the different treatments, a mix of Thermo Scientific cellular fluorescent dyes in PBS was added to each well for 30 min at 37◦C in a 5% CO_2_ incubator. This mix (Figs 4C and 7B) included Hoechst (1 μg/ml, nuclear (DNA) stain), MitoTracker Green (500 nM, potential‐independent mitochondrial stain), TMRE (500 μM, potential‐dependent mitochondrial stain), and Calcein‐AM Deep Red (0.5 μg/ml, cytosol stain). In Fig 7B, only lysosomes were stained with LysoTracker Deep Red (75 nM). Cells were then fixed with 4% paraformaldehyde (PFA), washed with PBS, and plates were transferred to an InCell2200 (GE Healthcare, U.K.) machine for image acquisition at 40× magnification. The output produced was based on comparative fluorescence intensity. Object segmentation was carried out using Multi‐target analysis in the GE analysis workstation to identify the nuclei and cell boundary. All the assay parameters (including the acquisition exposure times, objective, and the analysis parameters) were kept constant for all assay repetitions. For PAS staining of glycogen (Figs 4 and 6E), fixed cells were washed with PBS, permeabilized with 0.1% Triton X‐100, washed again stained, and then imaged.

### Target identification by nematic protein organization technique (NPOT)

NPOT^®^ was applied on human healthy fibroblasts and fibroblasts from two APBD patients. All the analyses were done by Inoviem Scientific Ltd. in a blinded manner. Protein homogenates from dry pellets of these fibroblasts were prepared by three cycles of fast freezing (liquid nitrogen) and slow thawing (on ice) and mixed at a maximal vortex speed for 30 s. Sample protein concentration was 50‐66 mg/ml as determined by the BCA method. NPOT^®^ is a proprietary technology offered by Inoviem Scientific dedicated to the isolation and identification of specific macromolecular scaffolds implemented in basic conditions or in pathological situations directly from human tissues. The technology is based on Kirkwood‐Buff molecular crowding (Zhou *et al*, 2008; Gee & Smith, 2009) and aggregation theory (Bloomfield, 2000; Shimizu, 2004). It enables the formation and label‐free identification of macromolecular complexes involved in physiological or pathological processes. The particular strength of Inoviem Scientific is the ability to analyze drug–protein and protein–protein interactions directly in human tissue, from complex mixtures without disrupting the native molecular conformation, consequently remaining in initial physiological or pathological condition.

Under laminar flow and sterile conditions, 10^‐6^ M of compounds 144DG11 and a negative control from our HTS screen (Solmesky *et al*, 2017) were mixed separately with the protein homogenates (containing soluble and membrane proteins) and subjected to NPOT^®^ isolation. The macromolecular assemblies associated with the ligand are separated using a differential microdialysis system, wherein the macromolecules (protein groups) migrate in the liquid phase based on their physico‐chemical properties. The migrating macromolecules gradually grow from nematic crystals to macromolecular heteroassemblies thanks to the molecular interactions between the tested drug and its targets. The heteroassemblies were left overnight and isolated in a 96‐well plate prior to identification by LC‐MS/MS.

The formed heteroassemblies in presence of 144DG11 and the negative control in APBD patients and HC fibroblasts are shown in Appendix Fig S2. Each compound in contact with the indicated protein homogenates gave rise to clearly‐defined heteroassemblies with common reticular morphology. The experiments were done in triplicate for each compound. For each of these biological replicates, heteroassemblies were isolated and their protein content analyzed by LC‐MS/MS. The negative control is obtained with the protein homogenate in the NPOT^®^ conditions without the addition of compound and does not present any aggregation. This further confirms that the formation of the heteroassembly is initiated by the compounds, and not by an endogenous small molecule, through their interactions with primary targets.

Under a Zeiss macroscope SteREO Discovery V8, each formed heteroassembly was isolated by microdissection and washed in acetone prior to solubilization in standard HBSS solution. Solubilized proteins were filtered through a 4‐15% mini‐PROTEAN gel. After migration, the gel was colored with a colloidal blue solution in order to visually estimate the number of proteins present in the gel, and the relative quantity of proteins to use for the following digestion step and injection in the LC‐MS/MS instrument for proteomics analysis.

Proteomics was outsourced to the “Laboratoire de Spectrométrie de Masse Bio‐Organique” (LSMBO) from the UMR 7178. Heteroassemblies were solubilized directly in 10 μl of 2D buffer (7 M Urea, 2 M Thiourea, 4% CHAPS, 20 mM DTT, 1 mM PMSF). Proteins were precipitated in acetate buffer and centrifuged for 20 min at 7500 g. Thereafter, pellets were digested for 1 h with Trypsin Gold (Promega) at 37°C. Trypsin Gold was resuspended at 1 μg/μl in 50 mM acetic acid, then diluted in 40 mM NH_4_HCO_3_ to 20 μg/ml. The samples were dried in Speed Vac^®^ at room temperature. Peptides were purified and concentrated by using ZipTip^®^ pipette tips (Millipore Corporation) before proceeding for mass spectrometry analysis through 1‐h nano‐LC‐MS/MS analyses protocol in an ESI‐QUAD‐TOF machine. Proteins were identified using Mascot software (Rank=1, score=25, minimal length=6 amino acids, FDR=1%). For peptide mapping, the following database was used: HumaniRTUN_DCpUN_JUS Bank (for human samples). For data analysis and target deconvolution Inoviem Scientific developed its own database and software to allow an accurate and robust analysis of the proteins present in NPOT^®^ datasets and simplify proteins ranking while removing protein contaminants. Inoviem Protein Ranking and Analysis (InoPERA^®^) database comprises all the NPOT^®^ datasets obtained on various tissues, organs or cell lines, varied species, and unrelated chemical compounds. InoPERA^®^ software is then able to calculate the occurrence of one given gene in the entire database, or specific datasets matching defined criteria of species, organs, etc. Inoviem removed contaminants that have been observed in NPOT^®^ performed in human tissues and cells, which correspond to 613 NPOT^®^ coupled LC‐MS/MS analyses. Consequently, this tool is able to quickly highlight rare proteins within a dataset that would make new therapeutic targets (Fig 5B).

Another bioinformatics resource—DAVID, was also used to find tissue‐specific expression, gene ontology, and functional‐related gene groups enrichment. Network enrichment within a dataset was investigated using STRING analysis (string‐db.org). STRING is one of the core data resource of ELIXIR (as Ensembl or UniProt are) which contains known and predicted protein–protein interactions. Inoviem has used the stringent parameters, keeping only the known interactions (“experimentally determined” and “curated databases” interaction sources). This allowed deciphering the protein–protein associations within a complex dataset, which further completed the DAVID pathway analysis. In addition, Reactome (reactome.org)—a free, open‐source, curated and peer‐reviewed pathway database, was used. This database provides intuitive bioinformatics tools for the visualization, interpretation, and analysis of pathway knowledge to support findings obtained elsewhere.

In the bioinformatic pipeline, the first step of filtering consisted of removing the mass spectrometry “false positives”, i.e., the proteins found in one replicate and with only one specific peptide. Then, the datasets were compared in a 2 by 2 matrix (144DG11 and its respective negative control) in human skin fibroblast tissue. The next step of protein list analysis was identification of non‐specific proteins, *i.e*., proteins that are found in a recurrent manner in all NPOT^®^ experiments (InoPERA^®^). Contaminants (or “frequent hits”) observed in human skin fibroblasts were removed. Cleared proteins lists of the interactome thus represent potential specific targets for 144DG11. Using this pipeline, 28 proteins were found to interact specifically with 144DG11. 144DG11 interactome’s specific protein lists were then analyzed independently by DAVID to find tissue‐specific expression, gene ontology, and functional‐related gene groups enrichment. The main canonical and disease and function pathways underlying the 144DG11 interactome were the lysosomal membranes (reference: GO:0005765 and KEGG pathway hsa04142). In parallel, STRING analysis (string‐db.org) was used to visualize prominent nodes and enriched networks. For this first ranking of the compound interactome’s specific proteins, we did not use the signal intensity of the peptides sequenced by MS because 1) the intrinsic properties of the technology cannot be based on protein quantitation (conversely to classic immunoprecipitation protocol for example), and 2) we do not use LC‐MS/MS quantitative protocols (which would imply higher cost and longer time analysis). This unbiased analysis allowed Inoviem to classify potential relevant proteins and categorize them according to their involvement in specific pathways, or in relation with specific diseases. Following this bioinformatic selection, 8 proteins belonging to the autophagosomal‐autolysosomal pathway were discovered (Fig 5B). The discovery of this well‐defined and enriched network demonstrates the overall success of the NPOT^®^ experiment.

### Western immunoblotting

Soluble protein factions were mixed with sample buffer and a reducing reagent (Life Technology) and warmed for 10 min at 70°C for protein denaturation. Samples were loaded on 10% Bis‐Tris gels or 4–12% gradient gels (Invitrogen) with PrecisionPlus^®^ Pre‐stained Protein Standard protein ladder (Bio‐rad) in MES SDS running buffer (Life Technology). Samples were run for 25‐45 min at 200V in the Mini Gel Tank 21 apparatus (Life Technology). Protein transfer onto nitrocellulose membrane was preform in iBlot^®^ 2 Gel Transfer Device (Life Technology). Membranes were then incubated for 1 h in 5% BSA (bovine serum albumin) in Tris‐buffered saline with Tween 20 (TBST, pH=8) blocking solution for 1h. Western blot (WB) primary antibodies (see below) were diluted in WB blocking solution and then added for overnight incubation at 4 °C. Blots were probed with HRP‐conjugated secondary antibodies (see below) diluted in WB blocking solution for 1h at room temperature, followed by three TBST washes. To detect protein bands, ECL femto‐kit (CYANAGEN) was used for development of membranes and signal detection was performed by the Amersham Imager 600 (Danyel Biotech). Quantification of band intensities was performed by densitometry analysis using ImageJ (Research Services Branch).

### List of primary antibodies (all applied at 1:1,000 titer, unless indicated otherwise)

LC3 from rabbit (Cell signaling, applied at 1:200 for immunohistochemistry detected by immunofluorescence (IHC‐IF)), HEXA from rabbit (Abcam), LAMTOR4 from rabbit (Cell signaling), LIMP‐2 from rabbit (Abcam), GAA from rabbit (Abcam), GBA from rabbit (Abcam), Actin‐HRP from mouse (Abcam), LAMP1 from rabbit (Abcam, applied at 1:50 for IHC‐IF), p62 from rabbit (Abcam, applied at 1:400 for IHC‐IF). *Secondary antibodies*: HRP‐conjugated donkey anti‐rabbit and anti‐mouse, or, for IHC‐IF, Alexa‐Fluor 488‐conjugated goat anti‐mouse Abs (Abcam). *Cellular thermal shift assay (CETSA)*. Cell lysates were treated with 50 uM compound A, or an equivalent volume of DMSO, and then heated to generate a melt curve. Precipitated (denatured) protein and cellular debris were separated by centrifugation, and the soluble fraction was subjected to SDS‐PAGE and immunoblotted with the antibodies indicated in Fig 5 (see (Jafari *et al*, 2014) for details).

### Surface plasmon resonance (SPR) assays to test 144DG11 binding to LAMP1

Luminal N‐terminus (aa 29‐382) of bioactive LAMP1 (binds Galectin‐3 with apparent K_D_ < 25 nM) with post‐translational modifications (R&D systems (4800‐LM‐050)) was reconstituted at 200 µg/ml in PBS and immobilized to a gold‐carboxymethylated dextran sensor chip by direct amine coupling. Sensogram experiments consisting of association and dissociation at the concentration ranges and pH values indicated in Fig 5 were then conducted. Sensogram signal (Fig 5) was normalized by reference surface and buffer signal subtraction.

### LAMP1 deglycosylation

Mature human LAMP1 consists of a 354‐amino acid (aa) luminal domain, a 23 aa transmembrane segment, and a 12 aa cytoplasmic tail. Its luminal domain is organized into two heavily N‐glycosylated regions separated by a Ser/Pro‐rich linker that carries a minor amount of O‐linked glycosylation. Possible interaction between 144DG11 and a non‐glycosylated form of LAMP1 was tested in order to establish whether the observed slow k_on_ at pH 4.5 (calculated to be 1.31/millisecond, Fig. 5D) can be explained by diffusion interference of the heavy LAMP1 glycosylation. Deglycosylation was performed chemically by trifluoromethanesulfonic acid (TFMS) which completely removes all N‐ and O‐linked glycans while preserving the protein structure. For the deglycosylation experiments, 25 μg of glycosylated LAMP1 luminal part was dialyzed, lyophilized, and then deglycosylated using manufacturer’s recommendations (GlycoProfile™ IV Chemical Deglycosylation Kit, Sigma‐Aldrich). RNase B (provided in the deglycosylation kit) was used as a positive control for a glycosylated protein. Finally, after short (24h) or long (72h) dialysis, the glycosylation status of LAMP1 and RNase B was tested by 15% SDS‐PAGE mobility shift gel stained with QC colloidal Coomassie stain (#1610803, Bio‐Rad). Our results (Fig. 5E, Upper panel) demonstrate a full deglycosylation of LAMP1 luminal part, with a unique band appearing after a long dialysis. RNAse B was also efficiently deglycosylated confirming the successful completion of the experiment. Glycosylated native LAMP1 protein, produced in mouse myeloma cells, migrated at around 110 kDa (Fig 5E, lane 1), as expected from its multiple N‐ and O‐glycosylation status. Upon TFMS treatment (Fig 5E, lanes2 and 3), LAMP1 migrated at ≈ 40 kDa with no band at 110 kDa, indicating that 100% of the protein was efficiently deglycosylated. Long dialysis (Fig 5E, lanes 3 and 6) enriched the deglycosylated bands. Fig 5E, lower panel, shows a sensorgram showing the interaction between deglycosylated LAMP1‐Nter protein (degLAMP1‐Nt) and 144DG11 (OKMW‐XX1). degLAMP1‐Nt was immobilized on the sensor chip CM5 using an alternative protocol without ethanolamine (Chip 2) and 144DG11 was diluted in HBS‐EP pH 4 at the indicated concentrations, while the running buffer was at pH 5. The sensorgrams correspond to normalized signal (meaning subtracted of reference surface and buffer signal).

### Computational docking analysis

LAMP1 sequence is divided into six segments (Terasawa *et al*, 2016): 1. residues M1‐A28: signal sequence; 2. residues A29‐R195: N‐terminal domain; 3. residues P196‐S216: linker between the domains; 4. residues S217‐M382: C‐terminal domain; 5. residues E383‐V405: the transmembrane segment; residues G406‐I417: cytoplasmic domain. We analyzed only the N‐ and the C‐terminal domains since 1. the signal sequence, transmembrane segment, and the cytoplasmic domain are assumed to be irrelevant for the binding of small molecules; 2. the linker between the domains is unstructured and heavily glycosylated (7 out of 20 residues) and thus too complicated to model. We have not considered glycosylation in the N‐ and the C‐terminals. The C‐ and N‐terminal domains were modeled based on the known crystal structure of mouse LAMP1 C‐terminal domain (PDB ID 5gv0) which is structurally highly similar to the N‐terminal domain (Terasawa *et al*, 2016). The MODELLER software tool (Webb & Sali, 2016) was used for homology modeling, producing 5 optional models for each domain. The obtained 10 models (as well as 5gv0 itself) were prepared in pH 5 by the “protein preparation wizard” as implemented in Schrodinger 2020‐2. Possible binding sites were identified by three different computational tools: SiteMap (Halgren, 2009), FtSite (Kozakov *et al*, 2015) and fPocket (Le Guilloux *et al*, 2009). Overall, 130 optional sites were identified in 11 LAMP1 3D structures. Docking computations were performed for each of the putative binding sites: 418 out of a large and diverse database of ˜ 30 million molecules were chosen as decoys according to 144DG11 applicability domain (Lipinski rules properties). The decoys library was narrowed down to 233 based on chemical similarity (Tanimoto coefficient ≥ 0.7). Docking computations for 144DG11 in a set of molecules composed of 144DG11 and 233 decoys (prepared in pH 5) were performed for every putative binding site in every model (overall 130 sites). The computations were performed using the Glide algorithm (Friesner *et al*, 2006), as implemented in Schrodinger 2020‐2. According to the docking results analysis (Dataset EV2), in 18 out of 130 sites 144DG11 was ranked at the top 10% (rankings 1‐24, Dataset EV2). Analyzing the results, we realized that site 1 of SiteMap, site 3 of fPocket, and site 2 of FtSite refer to the same pocket (residues F50‐D55, N62, L67, F118, Y120‐L122, T125, L127‐S133, N164‐V166).

We examined the differences between the binding modes of 144DG11 to the common site as defined by each software tool (Fig 5F) and concluded that 144DG11 tends to bind in a similar binding mode regardless of the software by which the site was configured (two out of three binding modes (SiteMap and fPocket) were identical while in FtSite, part of the molecule went through a rotation relative to the other two, but overall, the poses were highly similar).

To ensure 144DG11 specificity to the identified binding site, we repeated the analysis presented above for all 233 decoys. MarvinSketch was used to test the chemical structures of these molecules (MarvinSketch 20.13.0, ChemAxon (https://www.chemaxon.com)). Only in 13 out of 233 molecules, we observed the same results as for 144DG11—i.e., the molecules were successfully docked to pockets predicted by all three tools. Moreover, the pocket identified for 144DG11 (Fig 5F) matched 4 molecules out of 13). From these results, we shall deduce that the molecule 144DG11 selectively binds LAMP1 at the identified binding site.

In summary, we have computationally identified a possible binding site for 144DG11 in the N‐terminal domain of LAMP1 and predicted with high certainty that this result is specific for 144DG11 since the probabilities to obtain similar results for decoy molecules are low.

### Transmission electron microscopy (TEM)

Liver tissue was minced and fixed in a solution containing 2% paraformaldehyde and 2.5% glutaraldehyde (EM grade) in 0.1 M sodium cacodylate buffer pH 7.3 for 2 h at RT, followed by 24 h at 4°C. Tissue was then washed 4 times with sodium cacodylate and postfixed for 1 h with 1% osmium tetroxide and 1.5% potassium ferricyanide in sodium cacodylate. Then, sample was washed 4 times with the same buffer and dehydrated with graded series of ethanol solutions (30, 50, 70, 80, 90, 95%) for 10 min each and then 100% ethanol 3 times for 20 min each. Subsequently, samples were treated with 2 changes of propylene oxide. Samples were then infiltrated with series of epoxy resin (25, 50, 75, 100% – 24 h in each) and polymerized in the oven at 60°C for 48 h. The blocks were sectioned by an ultramicrotome (Ultracut E, Riechert‐Jung), and obtained sections of 80 nm were stained with uranyl acetate and lead citrate. Sections were observed by Jeol JEM 1400 Plus Transmission Electron Microscope, and images were taken using Gatan Orius CCD camera.

*IHC‐IF* was done on deparaffinized sections (10 μm thick). Prior to staining, antigen retrieval was performed as follows: For LAMP1 and p62, section slides were soaked in 10 mM Tris/1 mM EDTA pH 9 buffer and heated at 90°‐100° in a pressure cooker for 20 min. For LC3, section slides were soaked in 10 mM sodium citrate pH 6 and similarly heated. Sections were then washed 3X 5 min in PBS 1X. Then, slides were incubated for 1h at RT in a blocking solution, consisting of 0.1% Triton X‐100, and 2.5% BSA in PBS, which was also used for diluting the Abs. Sections were incubated in primary Abs overnight (16 h) at 4°C. Sections were washed again 3X 5 min in PBS 1X and then incubated in the appropriate secondary Abs for 1 h at RT, washed again 3X 5 min in PBS, and counterstained with DAPI in mounting fluid (Antifade, Abcam). Stained sections were imaged by the Zeiss LSM710 AxioObserver confocal microscope using a PlanAchromat 63x/1.40 Oil DIC objective. Image quantification was performed by the ImageJ software.

### Lentiviral infection

Lentiviral particles hosting LAMP1 shRNA fused to EGFP, or EGFP alone control plasmids were mixed with 8 μg/ml polybrene and supplemented to 80% confluent fibroblasts in fresh full medium for 8 h. Following incubation with lentiviral particles, medium was replaced and cells were examined by an epifluorescent microscope for EGFP fluorescence, which was normally observed after 72 h, as an indication of the expression of the lentiviral construct. Assays were conducted within 24 h after EGFP fluorescence was observed. *Lysosomal pH* was determined in APBD primary fibroblasts transduced with lentiviruses encoding for GFP or GFP‐shLAMP1 (see Materials & Methods) and treated or not with 50 µM 144DG11 for 24 h. Transduced cells were stained with 5µM Lysosensor Yellow/Blue DND‐160 just before acquisition in a BD‐LSRII flow cytometer using the 375 nm laser line and the 505 LP and 450/50 BP filters to detect the yellow and blue emissions of Lysosensor, respectively. The 488 nm laser line with the 530/30 BP filter was used to detect GFP fluorescence. Yellow Lysosensor and GFP fluorescence were compensated. To confirm fluorescence is lysosomal, cells treated with Lysosensor were also analyzed by the Nikon A1R confocal microscope using the 488 nm and 405 nm laser lines for exciting GFP and Lysosensor (only yellow fluorescence), respectively. See Materials & Methods for more details on experimental procedures.

### Proteomics

#### Sample preparation for MS analysis

Cell lysates in RIPA buffer containing protease inhibitors were clarified by centrifugation (Fig 7), and 40 μg of protein was used for protein precipitation by the chloroform/methanol method (Wessel & Flügge, 1984). The precipitated proteins were solubilized in 100 μl of 8 M urea, 10 mM DTT, 25 mM Tris‐HCl pH 8.0 and incubated for 30 min at 22°C. Iodoacetamide (55 mM) was added, and samples were incubated for 30 min (22°C, in the dark), followed by addition of DTT (10 mM). Fifty μl of the samples was transferred into a new tube, diluted by the addition of 7 volumes of 25 mM Tris‐HCl pH 8.0, and sequencing‐grade modified trypsin (Promega Corp., Madison, WI) was added (0.35 μg/sample) followed by incubation overnight at 37°C with gentle agitation. The samples were acidified by addition of 0.2% formic acid and desalted on C18 home‐made Stage tips. Peptide concentration was determined by absorbance at 280 nm, and 0.75 μg of peptides was injected into the mass spectrometer.

#### nano‐LC‐MS/MS analysis

MS analysis was performed using a Q Exactive‐HF mass spectrometer (Thermo Fisher Scientific, Waltham, MA USA) coupled on‐line to a nanoflow UHPLC instrument, Ultimate 3000 Dionex (Thermo Fisher Scientific, Waltham, MA USA). Peptides dissolved in 0.1% formic acid were separated without a trap column over a 120 min acetonitrile gradient run at a flow rate of 0.3 μl/min on a reverse phase 25‐cm‐long C18 column (75 μm ID, 2 μm, 100 Å, Thermo PepMapRSLC). The instrument settings were as described in Scheltema *et al* (2014). Survey scans (300–1,650 m/z, target value 3E6 charges, maximum ion injection time 20 ms) were acquired and followed by higher energy collisional dissociation (HCD)‐based fragmentation (normalized collision energy 27). A resolution of 60,000 was used for survey scans, and up to 15 dynamically chosen most abundant precursor ions, with “peptide preferable” profile, were fragmented (isolation window 1.6 m/z). The MS/MS scans were acquired at a resolution of 15,000 (target value 1E5 charges, maximum ion injection times 25 ms). Dynamic exclusion was 20 sec. Data were acquired using Xcalibur software (Thermo Scientific). To avoid a carryover, the column was washed with 80% acetonitrile and 0.1% formic acid for 25 min between samples.

#### MS data analysis

Mass spectra data were processed using the MaxQuant computational platform, version 1.6.14.0. Peak lists were searched against the Uniprot human FASTA sequence database from May 19, 2020, containing 49,974 entries. The search included cysteine carbamidomethylation as a fixed modification, and N‐terminal acetylation and oxidation of methionine as variable modifications and allowed up to two miscleavages. The match‐between‐runs option was used. Peptides with a length of at least seven amino acids were considered, and the required FDR was set to 1% at the peptide and protein level. Relative protein quantification in MaxQuant was performed using the label‐free quantification (LFQ) algorithm (Cox *et al*, 2014). Statistical analysis (*n* = 4‐7) was performed using the Perseus statistical package (Tyanova *et al*, 2016). Only those proteins for which at least 3 valid LFQ values were obtained in at least one sample group were accepted for statistical analysis by t‐test (*P* < 0.05).

### Statistics

In general, sample size was at least *n* = 3 for the *ex vivo* experiments and *n* = 7 for the *in vivo* experiments. Based on the differences observed, these sample sizes were sufficient to provide the acceptable power of at least 80% (Charan & Kantharia, 2013). Blinding in *in vivo* studies was obtained by encoding the animals in the different treatment groups. To obtain randomization, litters from mice born a few days apart were pooled and pups of similar weight were divided into vehicle and 144DG11‐treated arms.

Specifically, in Fig 1, the significance of overall trends was tested by two‐way ANOVA with repeated measures. This test determines how a response is affected by two factors: 144DG11 *v* control, which is given repeatedly (hence repeated measures), and duration of administration. The Bonferroni test was used to compare between 144DG11 and vehicle in a way which corrects for the multiple comparisons and is therefore very robust (since the threshold for determining significance at each time point is reduced in a manner inversely proportional to the number of comparisons). Consequently, most differences at specific time points became insignificant due to the increase in the number of comparisons and sometimes we chose to also show the data of multiple t‐tests which do not correct for multiple comparisons. In Figs 4D and 6E, we used one‐way ANOVA with Sidak’s post hoc correction for multiple comparisons. Other statistical tests used were Student t‐tests.

### Study approval

*In vivo* work was approved by the Hebrew University IACUC. APBD patient‐derived skin fibroblasts were obtained under informed consent and approved by the Hadassah IRB committee.

## Author contributions

HV, KN, JD, MM, US, SW‐A, AD, YR, BD, HE, SB, KM, AP, and SS designed and performed experiments; HR, JT, HOA, and BM designed experiments and critically reviewed the manuscript; AL, and AM contributed clinical samples; OK and MW designed, supervised, and wrote the manuscript.

## Conflict of interest

Patent WO2018154578, awarded to OK and MW, pertains to 144DG11 results.

## For more information

For more information on APBD and on its research and promotion of clinical endeavors please consult the APBD Research Foundation at https://www.apbdrf.org/.

## Supporting information



Appendix

Expanded View Figures PDF

Dataset EV1

Dataset EV2

Movie EV1

Source Data for Figure 6A

## Data Availability

The datasets produced in this study are available in the following databases: Proteomics mass spectrometry data processed by the MaxQuant computational platform: ProteomeXchange Consortium with the dataset identifier PXD027583 (http://proteomecentral.proteomexchange.org/cgi/GetDataset?ID=pxd027583).
