# Supplementary material for: Alleviation of a polyglucosan storage disorder by enhancement of autophagic glycogen catabolism
Source: EMBO Mol Med. 2021 Sep 6;13(10):e14554. doi: 10.15252/emmm.202114554 (PMC8495453; doi:10.15252/emmm.202114554)
Supplement: Supplementary file 1 — Appendix [file EMMM-13-e14554-s004.pdf]

Appendix Kakhlon *et al.* (EMM-2021-14554)

Appendix Figures S1 to S4 with figure legends

Appendix Tables S1 to S2

Appendix Figures

Appendix Figure S1. An ADMET-incompatible compound (88095528 in Figure EV1) causing wounds in Gbe$^{ys/ys}$ mice.

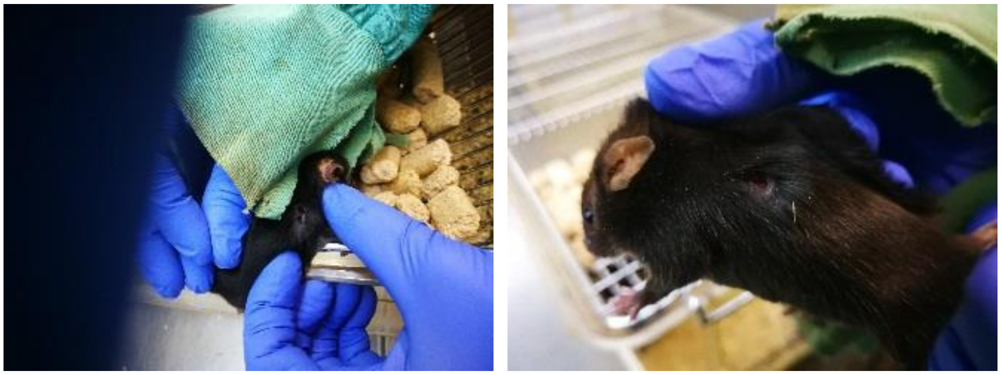

Appendix Figure S2. Heteroassemblies formed around 144DG11. Photos of the heteroassemblies (red circles) obtained by NPOT® on APBD-patient fibroblasts **(A)**, or HC fibroblasts **(B)** in the presence of compounds 144DG11
(OKMW-XX1) and OKMW-XXC (negative control) at 10^{-6} M. Each experiment was done in triplicate. Technical negative controls are obtained without the addition of any compound. Each picture represents a well of a 96-well plate.

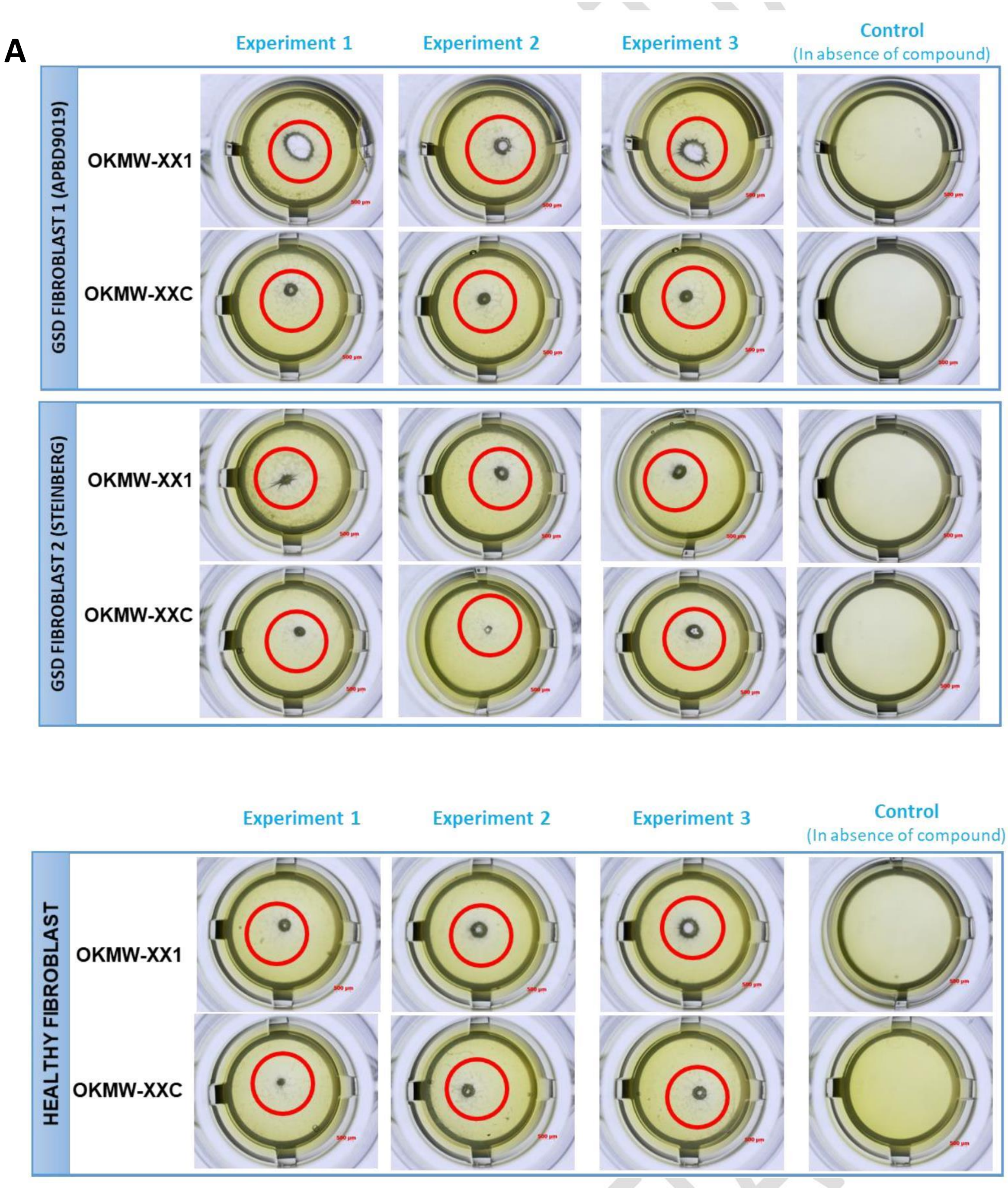

Appendix Figure S3. Endocytosis – a KEGG-Pathway enriched in APBD patient fibroblasts. Proteins up-regulated in APBD fibroblasts, as compared to HC (Figure 7E) were analyzed by the KEGG Pathway annotation tool in DAVID. The endocytosis pathway was found to be enriched in APBD fibroblasts ($p<1.10^{-6}$, $FDR<2.1x10^{-4}$). Stars denote proteins significantly modulated by the APBD diseased state.

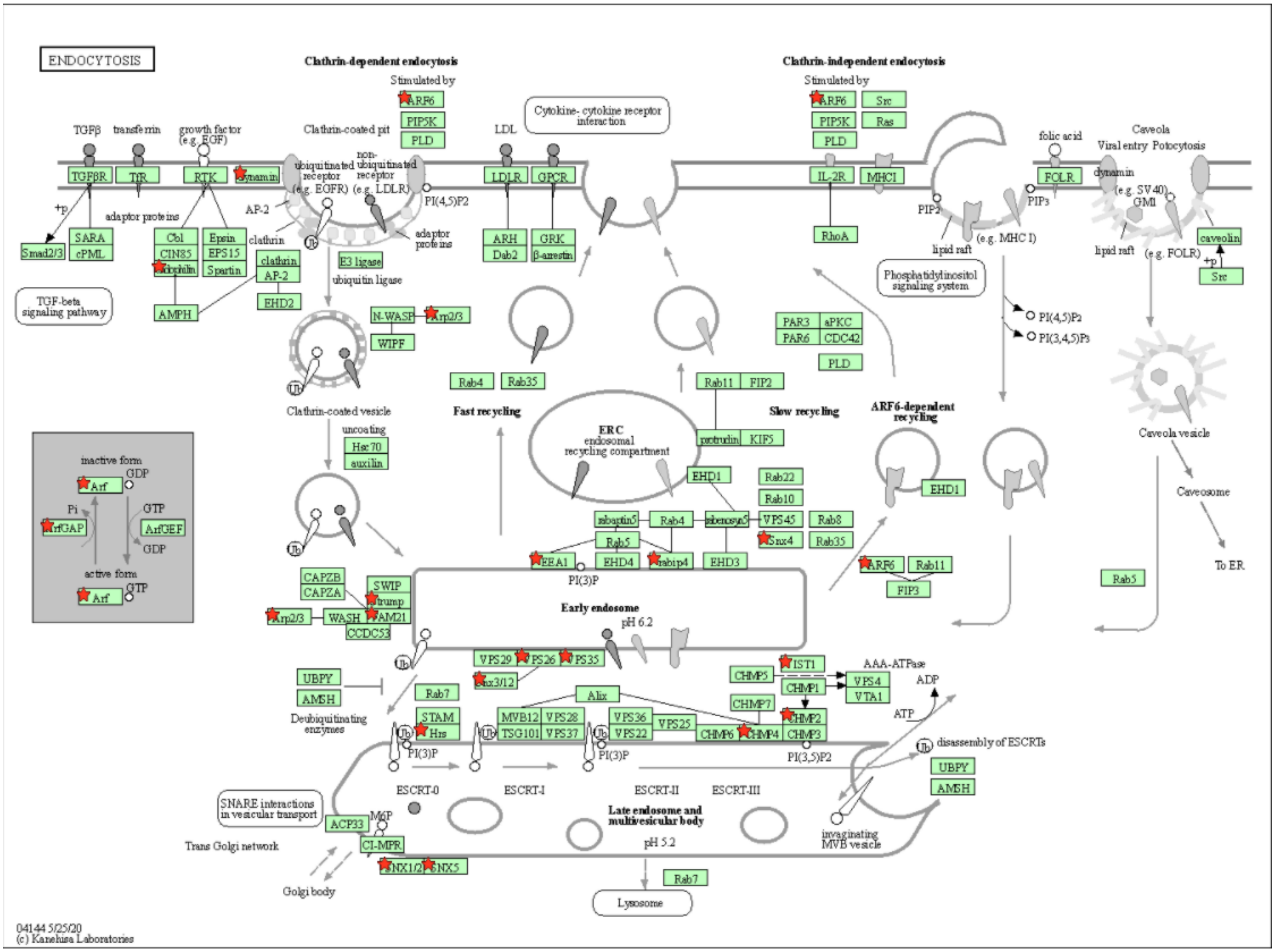

Appendix Figure S4. Oxidative Phosphorylation – a KEGG Pathway depleted in APBD fibroblasts. Proteins down-regulated in APBD fibroblasts, as compared to HC (Figure 7E) were analyzed by the KEGG Pathway annotation tool in DAVID. The oxidative phosphorylation pathway was found to be depleted in APBD fibroblasts ($p<1.9\times10^{-8}$, $FDR<1.5\times10^{-6}$). Stars denote proteins significantly down-modulated by the APBD diseased state.

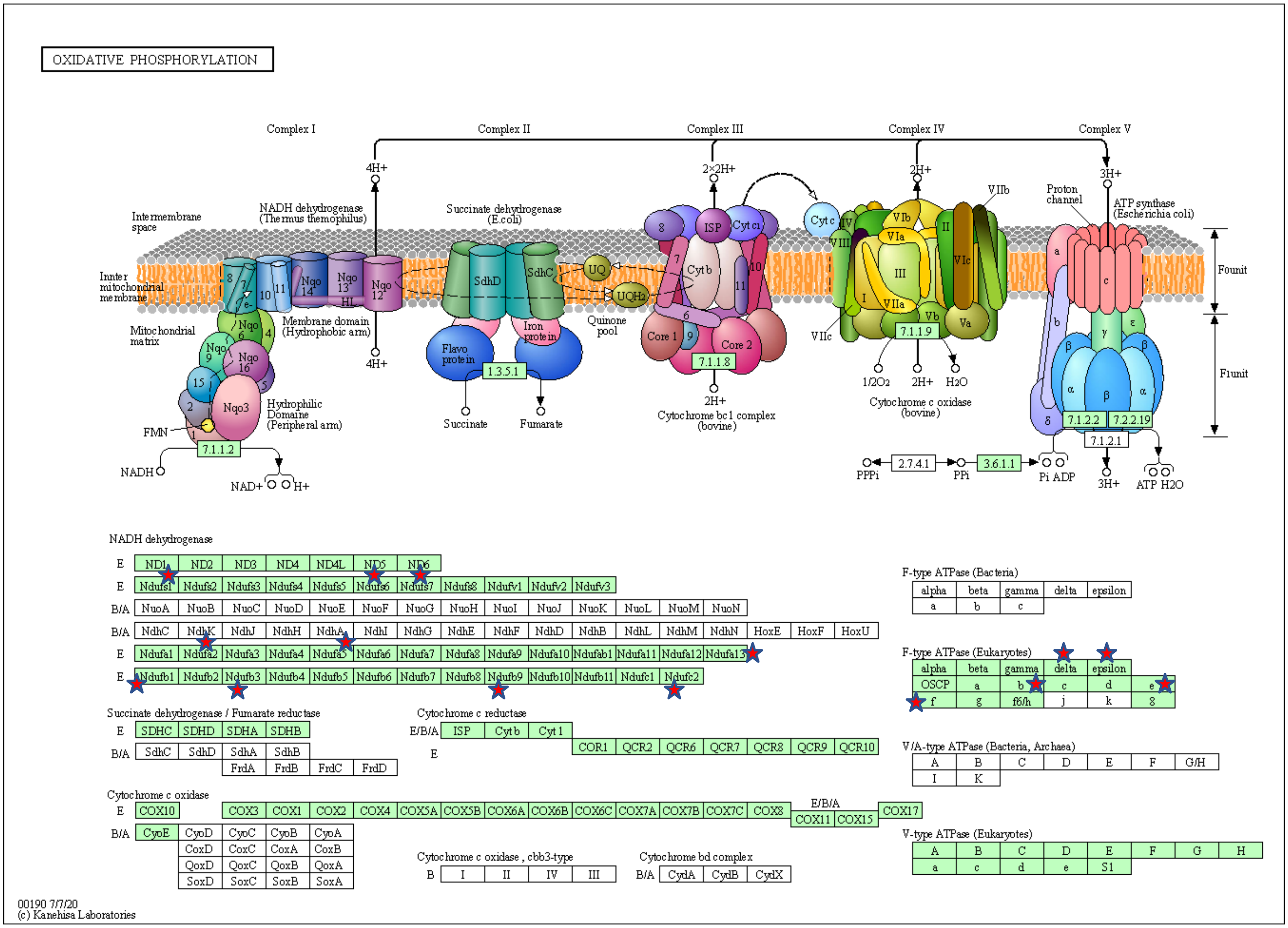

Appendix Table S1. Irwin test results of 144DG11.

	Vehicle	50 mg/kg 1h	250 mg/kg 1h	50 mg/kg 24h	250 mg/kg 24h
Coat color	Black	Black	Black	Black	Black
Presence of whiskers	3	3	3	3	3
Appearance of fur	2	2	2	2	2
Piloerection	0	0	0	0	0
Patches of missing fur on face	0	0	0	0	0
Patches of missing fur on body	0	0	0	0	0
Wounds	0	0	0	0	0
Transfer behavior	5	5	5	5	5
Body position	3.5	3.5	3	3.5	3
Tremor	0	0	0	0	0
Gait	0	0	0	0	0
Pelvic elevation	2	2	2	2	2
Tail elevation	1	1	1	1	1
Touch escape	2	2	2	2	2
Positional passivity	0	0	0	0	0
Trunk curl	0	0	0	0	0
Righting reflex	0	0	0	0	0
Salivation	0	0	0	0	0
Extension reflex	2	2	2	2	2

Irwin test scores: *Presence of whiskers* – 0 = None, 1 = A few, 2 = Most, but not a full set, 3 = A full set; *Appearance of fur* – 0 = Ungroomed and disheveled, 1 = Somewhat disheveled, 2 = Well-groomed (normal); *Piloerection* – 0 = None, 1 = Most hairs standing on end; *Patches of missing fur on face* – 0 = None, 1 = Some, 2 = Extensive; *Patches of missing fur on body* - 0 = None, 1 = Some, 2 = Extensive; *Wounds* – 0 = None, 1 = Signs of previous wounding, 2 = Slight wounds present, 3 = Moderate wounds present, 4 = Extensive wounds present; *Transfer behavior* – 0 = Coma, 1 = Prolonged freeze (>10 sec) then slight movement, 3 = Brief freeze (a few seconds) then active movement, 4 = Momentary freeze then swift movement, 5 = No freeze immediate movement, 6 = Extremely excited (manic); *Body position* – 0 = Completely flat (on stomach), 1 = Lying on side, 2 = Lying on back, 3 = Sitting or standing, 4 = Rearing on hind legs, 5 = Repeated vertical leaping; *Tremor* – 0 = None, 1 = Mild, 2 = Marked; *Gait* – 0 = Normal, 1 = Fluid but abnormal, 2 = Limited movement only, 3 = Incapacity; *Pelvic elevation* – 0 = Markedly flattened, 1 = Barely touches, 2 = Normal (3 mm elevation), 3 = Elevated (more than 3 mm elevation); *Tail elevation* – 0 = Dragging, 1 = Horizontally extended, 2 = Elevated (Straub tail); *Touch escape* – 0 = No response, 1 = Mild (escape response to firm stroke), 2 = Moderate (rapid response to light stroke), 3 = Vigorous (escape response to approach); *Positional passivity* – 0 = Struggles when restrained by tail, 1 = Struggles when restrained by neck (finger grip, not scruffed), 2 = Struggles when held supine (on back), 3 = Struggles when restrained by hind legs, 4 = Does not struggle; *Trunk curl* – 0 = Absent, 1 = Present; *Righting reflex* – 0 = No impairment, 1-10 = Number of seconds required to right; *Salivation* – 0 = None, 1 = Slight margin of sub-maxillary area, 2 = Wet zone entire sub-maxillary area; *Extension reflex* – 0 = Severe defect in hind limb extension reflex, 1 = Mild defect in hind limb reflex, 2 = Normal.

Appendix Table S2. Decoy molecules docked to pockets predicted by 3 computational tools.

Thirteen decoys were successfully docked to several pockets, predicted by all three tools. SiteMap, FtSite, and fPocket.

Model, Site ranking	Compounds (InchiKeys)	Number of compounds
**5gv0, site112**	ISNBQFVIRRXMNN-UHFFFAOYSA-N	1
**HN.B99990001, site113**	SDIOOFTYDJNAOO-UHFFFAOYSA-N	1
**HN.B99990004, site123**	**144DG11**<br>AFRQZZBRQMVSOV-UHFFFAOYSA-N*<br>OYOBHSNWLQNJLP-UHFFFAOYSA-N<br>ULMGNNFJGOAZQX-UHFFFAOYSA-N<br>VODWQUHDWRIEOI-UHFFFAOYSA-N	5
**HN.B99990005, site121**	LHKJBSUPOYJYCL-UHFFFAOYSA-N	1
**HN.B99990005, site233**	UIVJRUWFXCGSSM-UHFFFAOYSA-N<br>XDGVKHPNDVKOPJ-UHFFFAOYSA-N	2
**HC.B99990001, site223**	OHHMZSKNHGGUAX-UHFFFAOYSA-N	1
**HC.B99990002, site131**	CKHGBJKQAHAIDZ-UHFFFAOYSA-N<br>GLXDFBGZVSRFGI-UHFFFAOYSA-N<br>WTTMQZWWJFRCSI-UHFFFAOYSA-N	3
**HC.B99990003, site233**	AFRQZZBRQMVSOV-UHFFFAOYSA-N*	1

* Note: this molecule binds to two different binding sites, indicating is probably promiscuous.
