# Supplementary material for: Alleviation of a polyglucosan storage disorder by enhancement of autophagic glycogen catabolism
Source: EMBO Mol Med. 2021 Sep 6;13(10):e14554. doi: 10.15252/emmm.202114554 (PMC8495453; doi:10.15252/emmm.202114554)
Supplement: Supplementary file 2 — Expanded View Figures PDF [file EMMM-13-e14554-s002.pdf]

Expanded View Figures

	Candidate		# of problems per program			Decission
			QikProp	SwissADME	AdmetSAR	
	01-02	86282818	4	3	5	
	03-06	15607447	4	4	6	
	07	42992072	1	2	5	
	08-09	25760823	4	1	4	
	10-11	82320451	2	0	5	
	12	42459198	1	2	4	
	13-14	37867671	1	2	5	
144-DG-11 →	15-16 [A]	27686904	2	1	3	Preferred
	17-18	17057751	2	2	5	
	19	38058095	2	0	2	Preferred
	20-23	36585388	4	2	6	
	24 [B]	88095528	3	1	5	
	25	57540036	1	4	4	
	26	54056378	4	2	4	
	27-28	83101459	2	2	3	Preferred
	29	76195865	1	0	3	Preferred
	30	34834825	3	1	4	
	31-32	68349003	3	1	4	
	33-36	78653061	3	1	6	

Figure EV1. ***In silico*** **ADMET (Absorption, Distribution, Metabolism, and Excretion Toxicity)-compatible, polyglucosan lowering compounds.**

Heatmap shows analysis of three different ADMET algorithms. On the left are ordinal numbers of the compounds according to the hits discovered in Solmesky *et al* (2017). A range of numbers refers to enantiomers. The second column from left is the ChemBridge catalog number of the compound, and the heatmap (level of green) demonstrates the number of violations predicted by each algorithm.

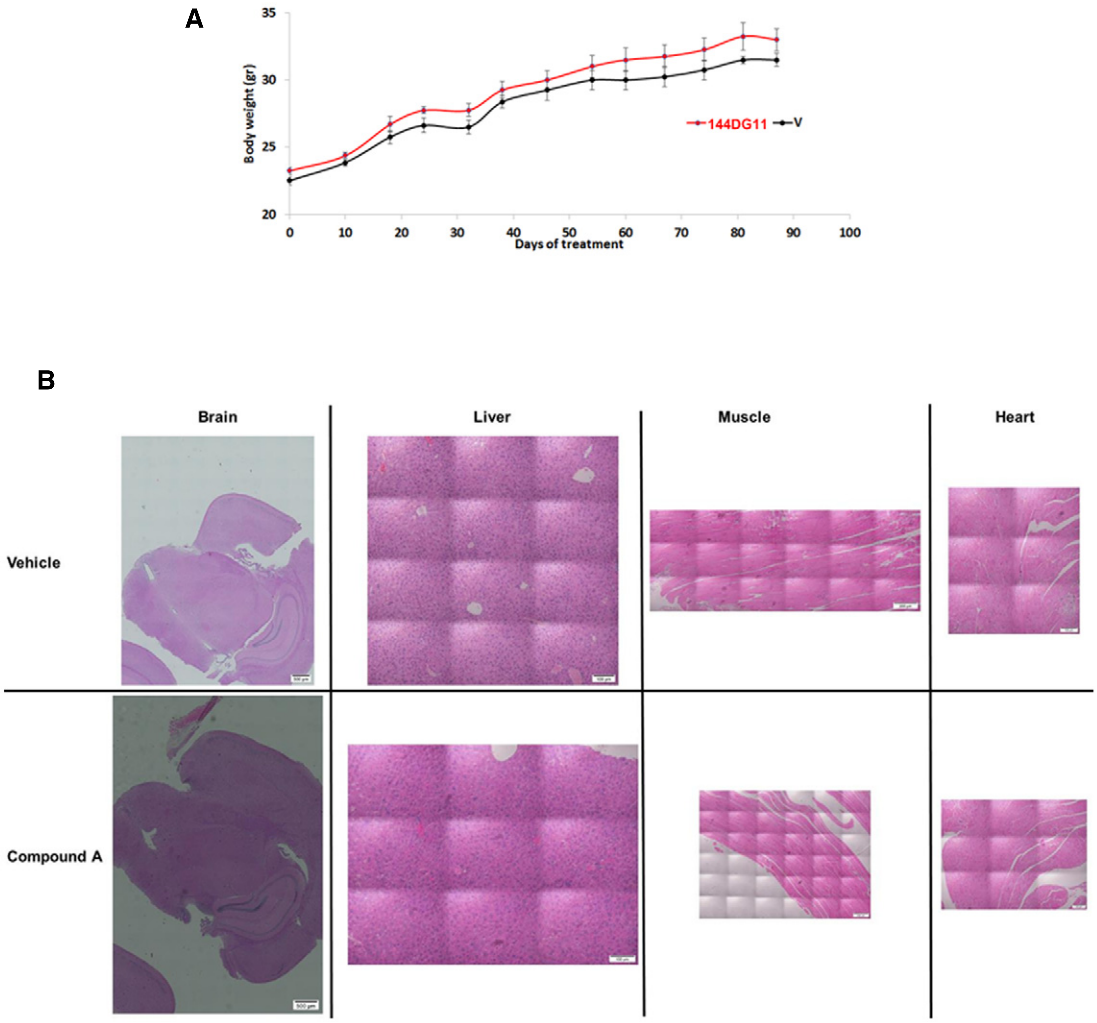

Figure EV2. Safety of 144DG11 in mice.

A Body weights of wild-type C57Bl6J mice treated with 144DG11 for 3 months. Mice were injected twice a week with 150 µl of 144DG11 at 250 mg/kg in 5% DMSO (red, *n* = 5), or an equal volume of 5% DMSO (V, vehicle) control (black, *n* = 6). Injections were intravenous for the first month and then subcutaneous for the following 2 months. No significant change between the two treatments is observed. Error bars represent S.D.

B Histology of 144DG11 tissues compared to vehicle control. Brain, liver, skeletal muscle, and heart slices of wild-type C57Bl6J mice treated for 3 months with 144DG11 as in (A). The slices were stained by H&E staining in order to visualize lesions. No lesions were apparent in either treatment. Scale bars, 500 µm (brain), 100 µm (liver), 200 µm (muscle), 100 µm (heart).

 © 2021 The Authors

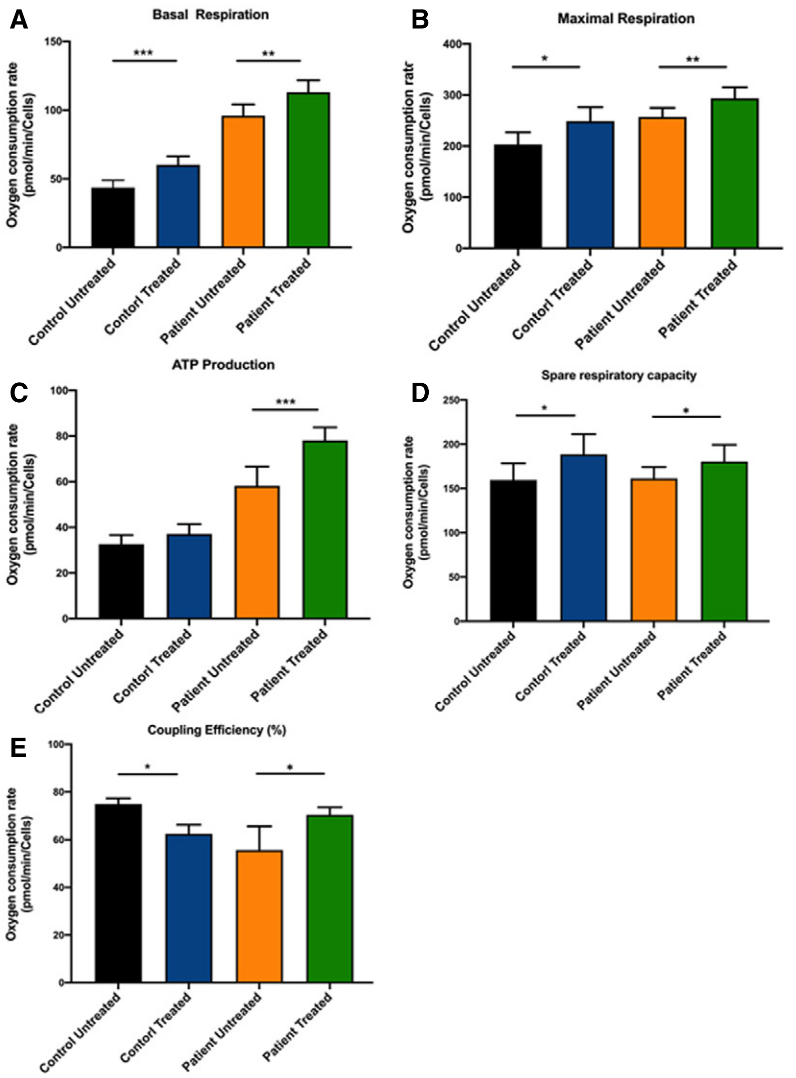

Figure EV3. Bioenergetic parameters of 144DG11-treated cells.

A Basal respiration in the indicated groups calculated as the mean OCR from the initiation of the experiment until first injection of oligomycin. 144DG11 led to a significant increase in basal respiration in both healthy control (***$P < 0.007$) and APBD patient (**$P < 0.02$) cells.

B Maximal respiration, defined as the difference between OCR values after FCCP and rotenone/antimycin supplementations, is increased by 144DG11 in both HC (*$P < 0.04$) and APBD patient (**$P < 0.03$) cells.

C ATP production, defined as the difference in OCR values between basal and post oligomycin levels, is increased by 144DG11 only in APBD patient cells (***$P < 0.006$).

D Spare respiratory capacity, defined as the difference between maximal (post FCCP) OCR and basal OCR values, was slightly (not significantly) increased by 144DG11 in both HC (*$P < 0.1$) and APBD patient (*$P < 0.13$) cells.

E Coupling efficiency, defined as the quotient (OCR following Oligomycin)/(Basal OCR), was increased by 144DG11 in HC (*$P < 0.01$) and APBD patient (*$P < 0.02$) cells.

Data information: All analyses are based on mean values of $n = 3$ biological replicates ± SEM. Statistical analysis was done by one-way ANOVA with Dunnett's post hoc tests.

© 2021 The Authors

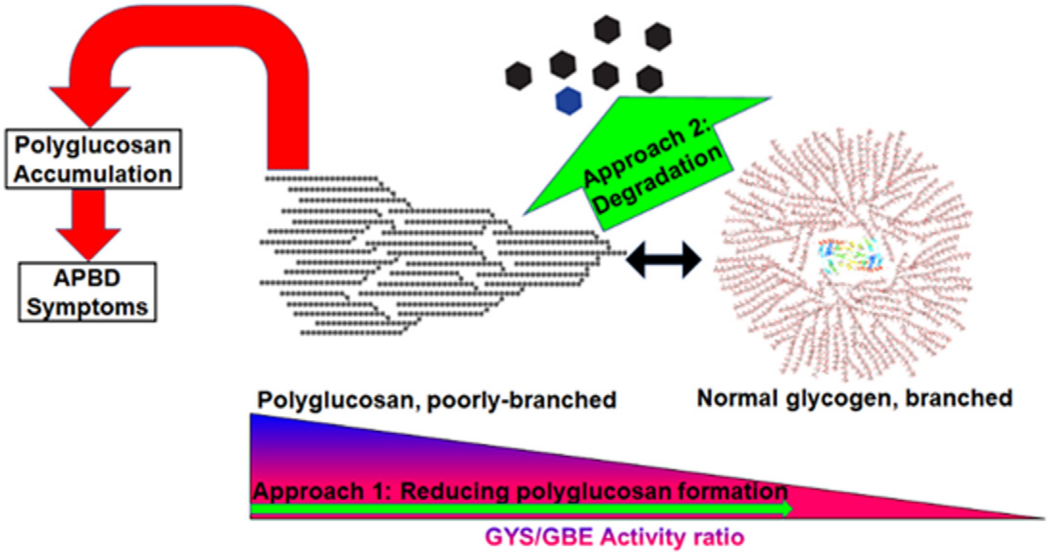

Figure EV4. Therapeutic strategies for APBD.

Therapeutic approaches for APBD are based on reduction of the GYS/GBE activity ratio or on direct PG and glycogen degradation. Blue hexagon, glucose; black hexagon, glucose-1-phosphate.

 © 2021 The Authors

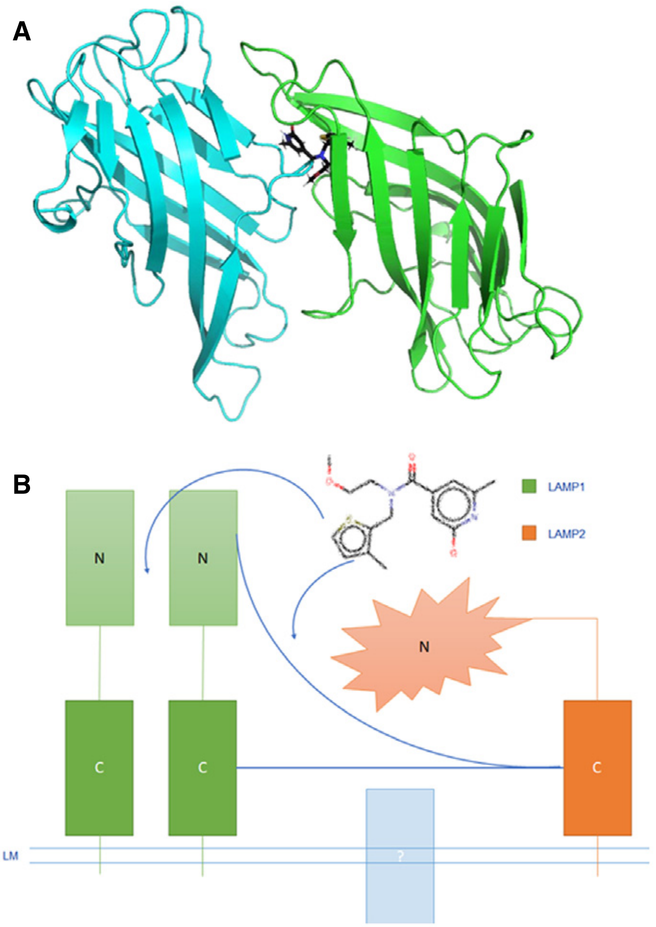

Figure EV5. 144DG11 at the LAMP1:LAMP1 interface.

To evaluate the probability that 144DG11 can interfere with LAMP1:LAMP1 interactions by binding to the predicted binding site at the LAMP1 N-terminus, we performed LAMP1 N-terminus:LAMP1 N-terminus protein:protein docking computations (A). According to the three highest ranked solutions (the top ranked result is shown in (A)), 144DG11 putative binding site is located in the LAMP1:LAMP1 interface. The possibility that 144DG11 also inhibits LAMP1: LAMP2 interactions requires additional computations.

A Predicted binding site for 144DG11 in LAMP1's N-terminal domain. Top ranked solution obtained by PATCHDOCK and FireDock servers. 144DG11, represented by black sticks, was docked to the putative binding site. LAMP1 N-terminal chains are represented in green and cyan.

B Schematic of the lysosomal membrane (LM), LAMP1, LAMP2, and the potential inhibitor 144DG11, a possible ancillary membrane protein mediating LAMP1 interaction.
