# Supplementary material for: Alleviation of a polyglucosan storage disorder by enhancement of autophagic glycogen catabolism
Source: EMBO Mol Med. 2021 Sep 6;13(10):e14554. doi: 10.15252/emmm.202114554 (PMC8495453; doi:10.15252/emmm.202114554)
Supplement: Supplementary file 5 — Movie EV1 [file EMMM-13-e14554-s007.zip › Movie EV1 legend.docx]

**Movie EV1: Motor effects of 144DG11.** The movie demonstrates the motor effects of 144DG11, administered at 250 mg/kg from the age of 4 months, in 8.5 month old Gbe^ys/ys^ (transgenic) male and female mice, as compared to their vehicle treated counterparts.
