# Supplementary figures and images for: Alleviation of a polyglucosan storage disorder by enhancement of autophagic glycogen catabolism
Source: EMBO Mol Med. 2021 Sep 6;13(10):e14554. doi: 10.15252/emmm.202114554 (PMC8495453; doi:10.15252/emmm.202114554)

Full unedited gels for Fig. 6A.

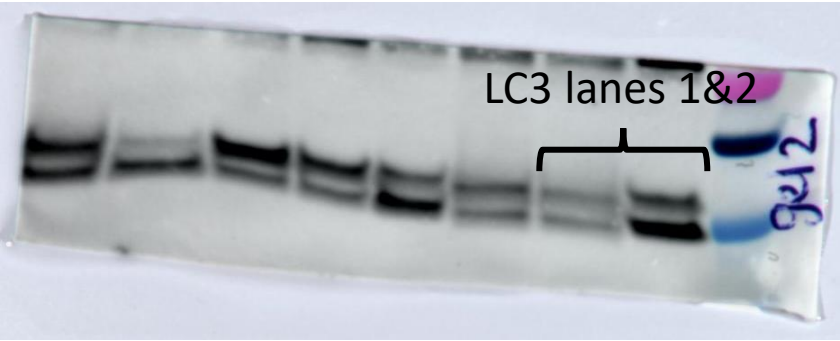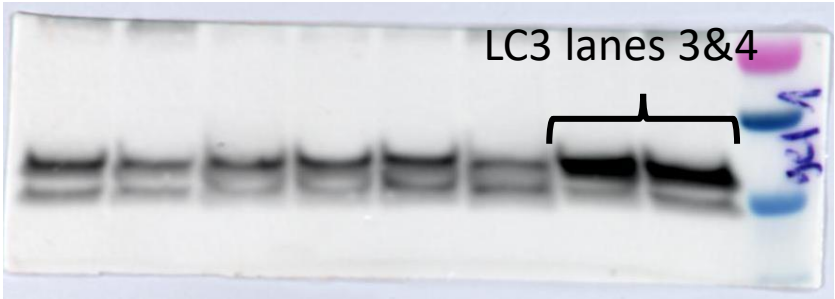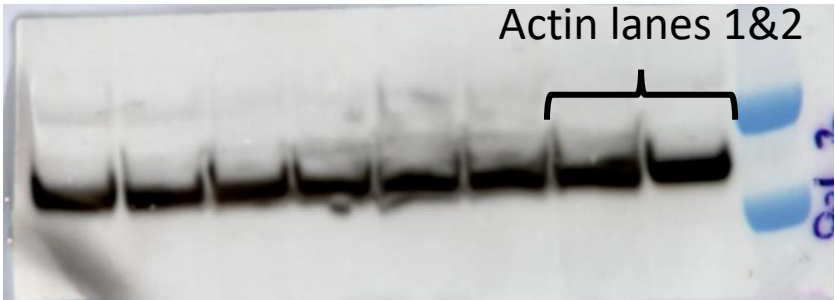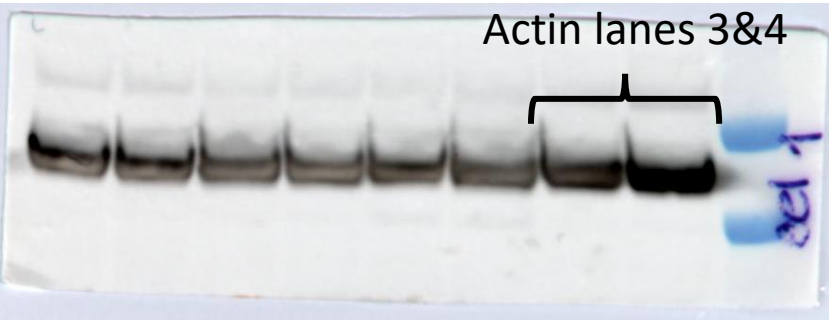

Supplement: Supplementary file 6 — Source Data for Figure 6A [file EMMM-13-e14554-s006.pdf]
