# Supplementary material for: Alleviation of a polyglucosan storage disorder by enhancement of autophagic glycogen catabolism
Source: EMBO Mol Med. 2021 Sep 6;13(10):e14554. doi: 10.15252/emmm.202114554 (PMC8495453; doi:10.15252/emmm.202114554)
Supplement: Supplementary file 7 [file EMMM-13-e14554-s005.pdf]

EMBO Molecular Medicine

Alleviation of a polyglucosan storage disorder by enhancement of autophagic glycogen catabolism

Or Kakhlon, Hilla Vaknin, Kumudesh Mishra, Jeevitha D'Souza, Monzer Marisat, Uri Sprecher, Shane Wald-Altman, Anna Dukhovny, Yuval Raviv, Benny Da'adoosh, Hamutal Engel, Sandrine Benhamron, Keren NItzan, Sahar Sweetat, Anna Permyakova, Anat Mordechai, Hasan Akman, Hanna Rosenmann, Alexander Lossos, Joseph Tam, Berge Minassian, and Miguel Weil
DOI: 10.15252/emmm.202114554

Corresponding author: Or Kakhlon (ork@hadassah.org.il)

Review Timeline:

Submission Date:	13th May 21
Editorial Decision:	2nd Jun 21
Revision Received:	3rd Aug 21
Editorial Decision:	9th Aug 21
Revision Received:	12th Aug 21
Accepted:	13th Aug 21

Editor: Zeljko Durdevic

Transaction Report:

(Note: With the exception of the correction of typographical or spelling errors that could be a source of ambiguity, letters and reports are not edited. Depending on transfer agreements, referee reports obtained elsewhere may or may not be included in this compilation. Referee reports are anonymous unless the Referee chooses to sign their reports.)

1st Editorial Decision 2nd Jun 2021

Dear Dr. Kakhlon,

Thank you for the submission of your manuscript to EMBO Molecular Medicine. We have now received feedback from two of the three reviewers who agreed to evaluate your manuscript. Given that referee #2 will unfortunately not be able to return his/her report in a timely manner, and that both referees #1 and #3 are overall positive, we prefer to make a decision now in order to avoid further delay in the process. As you will see from the reports below, the referees acknowledge the interest of the study but also raise important concerns that should be addressed in a major revision.

Further consideration of a revision that addresses reviewers' concerns in full will entail a second round of review. EMBO Molecular Medicine encourages a single round of revision only and therefore, acceptance or rejection of the manuscript will depend on the completeness of your responses included in the next, final version of the manuscript. For this reason, and to save you from any frustrations in the end, I would strongly advise against returning an incomplete revision.

We would welcome the submission of a revised version within three months for further consideration. However, we realize that the current situation is exceptional on the account of the COVID-19/SARS-CoV-2 pandemic. Please let us know if you require longer to complete the revision.

I look forward to receiving your revised manuscript.

Yours sincerely,

Zeljko Durdevic
Editor
EMBO Molecular Medicine

***** Reviewer's comments *****

Referee #1 (Comments on Novelty/Model System for Author):

Adult Polyglucosan Body Disease (APBD) is a neurological disorder that affects multiple tissues. Currently there is no curative treatment for patients with APBD. Kakhlon et al. reported the effectiveness and possible mechanisms of action of the polyglucosan-reducing compound 144DG11 in APBD mice and APBD patient cells. This therapeutic approach has the potential to cure patients with APBD and other polyglucosan body diseases. The experiments were well designed and conducted and the manuscript was well written.

Referee #1 (Remarks for Author):

Adult Polyglucosan Body Disease (APBD) is a neurological disorder that affects multiple tissues. Currently there is no curative treatment for patients with APBD. Kakhlon et al. reported the effectiveness and possible mechanisms of action of the polyglucosan-reducing compound 144DG11 in APBD mice and APBD patient cells. This therapeutic approach has the potential to cure patients with APBD and other polyglucosan body diseases. The experiments were well designed and conducted and the manuscript was well written.

Comments:

1. Liver glycogen content varies greatly if the mice were not fasted. The authors need to indicate whether the mice were fasted prior to euthanization.
2. Figure 2 shows that 144DG11 had no apparent effect on muscle polyglucosan bodies. What muscle was examined? Did you look at different skeletal muscles as well as smooth muscle (e.g., bladder)?
3. In Figure 6E, the LAMP1 KO was probably a typo of LAMP1 KD.
4. Figure7D should be Figure 7E in the paragraph on page 13 "As shown in Figure 7D, under 48 h starvation 12.2% and 6.8% of the 2,898 proteins analyzed were respectively up and down modulated in APBD-patient as compared to HC cells. Interestingly, endocytosis, a pathway implicated in lysosomal biogenesis and function, is a KEGG pathway upmodulated in APBD cells (Figure S9), while oxidative phosphorylation is down-modulated in APBD cells (Figure S10). As an important control, GBE was indeed down-modulated in the APBD cells (Figure 7D)".
5. Last paragraph on page 17 "Importantly, lysosomal glycogen degradation takes place in parallel with its cytoplasmic degradation (37), and, specifically, in a GSDIV mouse model, which also models APBD in mice, overexpression of the lysosomal glycogenase α-glucosidase corrected pathology (38)." This is inaccurate description. Consider to revise to "... overexpression of human GDE by gene therapy corrected pathology in skeletal muscle, liver, and brain (38) and treatment with recombinant human lysosomal enzyme acid α-glucosidase alleviated disease in the liver (Yi et al., Alglucosidase

alfa treatment alleviates liver disease in a mouse model of glycogen storage disease type IV. Mol. Genet. Metab. Rep., 2016).

Referee #3 (Comments on Novelty/Model System for Author):

While the work carried out by this team concerns both in vivo and in vitro approaches which is a real added value, the murine model could be better exploited.

Referee #3 (Remarks for Author):

The authors tested both in vivo and in vitro a glycogen lowering therapeutic molecule - 144 DG11 - obtained by high throughput screening. In the first part, the molecule is tested in the Gbe ys / ys murine model. Motor performances were improved and a significant reduction in polyglucosan bodies were observed in the brain, liver, heart, and peripheral nerves except muscle. By using indirect calorimetry, they demonstrated a shift of the respiratory quotient towards carborhydrate catabolism. The putative mechanisms of action were then deciphered in skin fibroblasts from APBD patients.
The combination of the two approaches in vivo and in vitro is undoubtedly an added value. The data obtained in vitro appear to be well controlled and robust. The murine model could however be much better exploited, in particular with regard to the results obtained in vitro on autophagy, LAMP 1 and the lysosome.

Major recommendations :
In the mouse model of APBD (Gbe ys/ys), please investigate:
- the lysosomal compartment both in liver or heart and skeletal muscle from Gbe and wt mice by using immunohistochemistry against LAMP1 and by quantification of LAMP1positive-areas for example
- autophagy through LC3 and p62 for liver or heart and skeletal muscle by using immunohistochemistry against LC3 and by quantification of LC3 - vacuolated area positive or by Western-blot for example.

Please rephrase the in vivo results p5, P6 and p7. It is sometimes very confusing to understand at what age the mice were injected and at what age they were sacrificed, and the results obtained. A schematic representation of the experimental design would be appreciated as an additional figure.

******* Reviewer's comments *******

We thank the reviewers for their constructive criticisms. We have made all requested changes, which undoubtedly improved the quality of our manuscript. Please find below the respective comments our replies and references to the relevant manuscript modifications.

Referee #1 (Comments on Novelty/Model System for Author):

Adult Polyglucosan Body Disease (APBD) is a neurological disorder that affects multiple tissues. Currently there is no curative treatment for patients with APBD. Kakhlon et al. reported the effectiveness and possible mechanisms of action of the polyglucosan-reducing compound 144DG11 in APBD mice and APBD patient cells. This therapeutic approach has the potential to cure patients with APBD and other polyglucosan body diseases. The experiments were well designed and conducted and the manuscript was well written.

Referee #1 (Remarks for Author):

Adult Polyglucosan Body Disease (APBD) is a neurological disorder that affects multiple tissues. Currently there is no curative treatment for patients with APBD. Kakhlon et al. reported the effectiveness and possible mechanisms of action of the polyglucosan-reducing compound 144DG11 in APBD mice and APBD patient cells. This therapeutic approach has the potential to cure patients with APBD and other polyglucosan body diseases. The experiments were well designed and conducted and the manuscript was well written.

Comments:
1. Liver glycogen content varies greatly if the mice were not fasted. The authors need to indicate whether the mice were fasted prior to euthanization.

Thank you for this comment. We agree. Glycogen levels can change considerably depending on the feeding regime. The mice did not fast before their euthanasia. This is now mentioned under Methods, *In vivo experiments*, on p. 20.

2. Figure 2 shows that 144DG11 had no apparent effect on muscle polyglucosan bodies. What muscle was examined? Did you look at different skeletal muscles as well as smooth muscle (e.g., bladder)?

We investigated the quadriceps as a representative striated skeletal muscle. This skeletal muscle is usually investigated in APBD models as the disease is a neuromuscular disorder, which affects motor abilities. For instance, like us, Akman *et al* (2015) *Hum Mol Genet* 24: 6801–6810 and also Chown *et al* (2020) *Ann Clin Transl Neurol* 7: 2186-2198 have also examined polyglucosans in APBD mouse model only in skeletal and cardiac muscles. We now specified that the analysis was done on the quadriceps muscle under *Histological PG and glycogen determination* in the Appendix.

3. In Figure 6E, the LAMP1 KO was probably a typo of LAMP1 KD.

Correct. Thanks for pointing this out. We corrected that.

4. Figure7D should be Figure 7E in the paragraph on page 13 "As shown in Figure 7D, under 48 h starvation 12.2% and 6.8% of the 2,898 proteins analyzed were respectively up and down modulated in APBD-patient as compared to HC cells. Interestingly, endocytosis, a pathway implicated in lysosomal biogenesis and function, is a KEGG pathway upmodulated in APBD cells (Figure S9), while oxidative phosphorylation is down-modulated in APBD cells (Figure S10). As an important control, GBE was indeed down-modulated in the APBD cells (Figure 7D)".

Corrected.

5. Last paragraph on page 17 "Importantly, lysosomal glycogen degradation takes place in parallel with its cytoplasmic degradation (37), and, specifically, in a GSDIV mouse model, which also models APBD in mice, overexpression of the lysosomal glycogenase α-glucosidase corrected pathology (38)." This is inaccurate description. Consider to revise to "... overexpression of human GDE by gene therapy corrected pathology in skeletal muscle, liver, and brain (38) and treatment with recombinant human lysosomal enzyme acid α-glucosidase alleviated disease in the liver (Yi et al., Alglucosidase alfa treatment alleviates liver disease in a mouse model of glycogen storage disease type IV. Mol. Genet. Metab. Rep., 2016).

Corrected. We cited there the wrong reference by Yi *et al*. Thank you for bringing that to our attention.

Referee #3 (Comments on Novelty/Model System for Author):

While the work carried out by this team concerns both in vivo and in vitro approaches which is a real added value, the murine model could be better exploited.

Referee #3 (Remarks for Author):

The authors tested both in vivo and in vitro a glycogen lowering therapeutic molecule - 144 DG11 - obtained by high throughput screening. In the first part, the molecule is tested in the Gbe ys / ys murine model. Motor performances were improved and a significant reduction in polyglucosan bodies were observed in the brain, liver, heart, and peripheral nerves except muscle. By using indirect calorimetry, they demonstrated a shift of the respiratory quotient towards carborhydrate catabolism. The putative mechanisms of action were then deciphered in skin fibroblasts from APBD patients.
The combination of the two approaches in vivo and in vitro is undoubtedly an added value. The data obtained in vitro appear to be well controlled and robust. The murine model could however be much better exploited, in particular with regard to the results obtained in vitro on autophagy, LAMP 1 and the lysosome.

Major recommendations :
In the mouse model of APBD (Gbe ys/ys), please investigate:
- the lysosomal compartment both in liver or heart and skeletal muscle from Gbe and wt mice by using immunohistochemistry against LAMP1 and by quantification of LAMP1positive-areas for example
- autophagy through LC3 and p62 for liver or heart and skeletal muscle by using immunohistochemistry against LC3 and by quantification of LC3 - vacuolated area positive or by Western-blot for example.

We accept the reviewer comment and added in Figure 6 new panels (current Fig. 6C and 6G) analyzing LAMP1 and autophagy (LC3 and p62) in liver and skeletal muscle sections taken from 144DG11 and vehicle treated Gbe$^{ys/ys}$ mice. As opposed to skeletal muscle, 144DG11 was both biodistributed (Figure 2C) and effective (Figures 2A, 2B, and 6B) in the liver of Gbe$^{ys/ys}$ mice. Therefore, we compared the effects of 144DG11 on our fixed and deparaffinized muscle and liver sections derived from 144DG11 and vehicle treated Gbe$^{ys/ys}$ mice. As expected, LC3 and p62 levels were indeed reduced by 144DG11 only in liver, and not in muscle, sections. In addition, LAMP1 staining area was reduced by 144DG11 only in liver sections, possibly associated with the compound-mediated improvement of lysosomal function.

Please rephrase the in vivo results p5, P6 and p7. It is sometimes very confusing to understand at what age the mice were injected and at what age they were sacrificed, and the results obtained. A schematic representation of the experimental design would be appreciated as an additional figure.

Done. A scheme of the experimental design is now added in the new Figure 1K.

1st Revision - Editorial Decision 9th Aug 2021

Dear Dr. Kakhlon,

Thank you for the submission of your revised manuscript to EMBO Molecular Medicine. I am pleased to inform you that we will be able to accept your manuscript pending the following final amendments:

1) In the main manuscript file, please do the following:

- Correct/answer the track changes suggested by our data editors by working from the attached document.
- Reduce keywords to max. 5.
- Add callouts for Fig EV5.
- In M&M, a statistical paragraph that should reflect all information that you have filled in the Authors Checklist, especially regarding randomization, blinding, replication.
- In author contributions is KM referring to Keren Nitzan.
- Tables should be black and white.
- Please use the following format to report the accession number of your data:

The datasets produced in this study are available in the following databases:
[data type]: [full name of the resource] [accession number/identifier] ([doi or URL or identifiers.org/DATABASE:ACCESSION])

Please check "Author Guidelines" for more information.
https://www.embopress.org/page/journal/17574684/authorguide#availabilityofpublishedmaterial

2) Datasets: Include movie legend as a .doc file and zipp it with the movie file. Please correct the legend in Dataset EV2.
3) Appendix: Please correct figure and table nomenclature to "Appendix Figure S1" etc. and "Appendix Table S1", also in the main text. Move M&M to main manuscript file.
4) Synopsis:
- Synopsis image: Please resize the synopsis image to 550 px-wide x (250-400)-px high and submit it as a high-resolution jpeg file.
- Please check your synopsis text and image, revise them if necessary and submit their final versions with your revised manuscript. Particular attention should be given to grammar and syntax. Please be aware that in the proof stage minor corrections only are allowed (e.g., typos).
5) For more information: There is space at the end of each article to list relevant web links for further consultation by our readers. Could you identify some relevant ones and provide such information as well? Some examples are patient associations, relevant databases, OMIM/proteins/genes links, author's websites, etc...
6) As part of the EMBO Publications transparent editorial process initiative (see our Editorial at http://embomolmed.embopress.org/content/2/9/329), EMBO Molecular Medicine will publish online a Review Process File (RPF) to accompany accepted manuscripts. This file will be published in conjunction with your paper and will include the anonymous referee reports, your point-by-point response and all pertinent correspondence relating to the manuscript. Let us know whether you agree with the publication of the RPF and as here, if you want to remove or not any figures from it prior to publication. Please note that the Authors checklist will be published at the end of the RPF.
7) Please provide a point-by-point letter INCLUDING my comments as well as the reviewer's reports and your detailed responses (as Word file).

I look forward to reading a new revised version of your manuscript as soon as possible.

Yours sincerely,

Zeljko Durdevic

Zeljko Durdevic
Editor
EMBO Molecular Medicine

***** Reviewer's comments *****

Referee #1 (Remarks for Author):

Suitable for publication.

The authors performed the requested editorial changes.

2nd Revision - Editorial Decision 13th Aug 2021

We are pleased to inform you that your manuscript is accepted for publication and is now being sent to our publisher to be included in the next available issue of EMBO Molecular Medicine.

EMBO PRESS

YOU MUST COMPLETE ALL CELLS WITH A PINK BACKGROUND ➔

PLEASE NOTE THAT THIS CHECKLIST WILL BE PUBLISHED ALONGSIDE YOUR PAPER

Corresponding Author Name: Or Kakhlon (ORCID ID 0000-0001-7435-6454) & Miguel Weil (ORCID ID 0000-

Journal Submitted to: EMBO Molecular Medicine

Manuscript Number: EMM-2021-14554

Reporting Checklist For Life Sciences Articles (Rev. June 2017)

This checklist is used to ensure good reporting standards and to improve the reproducibility of published results. These guidelines are consistent with the Principles and Guidelines for Reporting Preclinical Research issued by the NIH in 2014. Please follow the journal's authorship guidelines in preparing your manuscript.

A- Figures

1. Data

The data shown in figures should satisfy the following conditions:

- the data were obtained and processed according to the field's best practice and are presented to reflect the results of the experiments in an accurate and unbiased manner.
- figure panels include only data points, measurements or observations that can be compared to each other in a scientifically meaningful way.
- graphs include clearly labeled error bars for independent experiments and sample sizes. Unless justified, error bars should not be shown for technical replicates.
- if n< 5, the individual data points from each experiment should be plotted and any statistical test employed should be justified
- Source Data should be included to report the data underlying graphs. Please follow the guidelines set out in the author ship guidelines on Data Presentation.

2. Captions

Each figure caption should contain the following information, for each panel where they are relevant:

- a specification of the experimental system investigated (eg cell line, species name).
- the assay(s) and method(s) used to carry out the reported observations and measurements
- an explicit mention of the biological and chemical entity(ies) that are being measured.
- an explicit mention of the biological and chemical entity(ies) that are altered/varied/perturbed in a controlled manner.

- the exact sample size (n) for each experimental group/condition, given as a number, not a range;
- a description of the sample collection allowing the reader to understand whether the samples represent technical or biological replicates (including how many animals, litters, cultures, etc.).
- a statement of how many times the experiment shown was independently replicated in the laboratory.
- definitions of statistical methods and measures:
  - common tests, such as t-test (please specify whether paired vs. unpaired), simple χ2 tests, Wilcoxon and Mann-Whitney tests, can be unambiguously identified by name only, but more complex techniques should be described in the methods section;
  - are tests one-sided or two-sided?
  - are there adjustments for multiple comparisons?
  - exact statistical test results, e.g., P values = x but not P values < x;
  - definition of 'center values' as median or average;
  - definition of error bars as s.d. or s.e.m.

Any descriptions too long for the figure legend should be included in the methods section and/or with the source data.

In the pink boxes below, please ensure that the answers to the following questions are reported in the manuscript itself. Every question should be answered. If the question is not relevant to your research, please write NA (non applicable). We encourage you to include a specific subsection in the methods section for statistics, reagents, animal models and human subjects.

USEFUL LINKS FOR COMPLETING THIS FORM

http://www.antibodypedia.com
http://1degreebio.org
http://www.equator-network.org/reporting-guidelines/improving-bioscience-research-repor
http://grants.nih.gov/grants/olaw/olaw.htm
http://www.mrc.ac.uk/Ourresearch/Ethicsresearchguidance/Useofanimals/index.htm
http://ClinicalTrials.gov
http://www.consort-statement.org
http://www.consort-statement.org/checklists/view/32-consort/66-title
http://www.equator-network.org/reporting-guidelines/reporting-recommendations-for-tumo
http://datadryad.org
http://figshare.com
http://www.ncbi.nlm.nih.gov/gap
http://www.ebi.ac.uk/ega
http://biomodels.net/
http://biomodels.net/miriam/
http://jjj.biochem.sun.ac.za
https://osp.od.nih.gov/biosafety-biosecurity-and-emerging-biotechnology/
http://www.selectagents.gov/

B- Statistics and general methods

Please fill out these boxes ➔ (Do not worry if you cannot see all your text once you press return)

1.a. How was the sample size chosen to ensure adequate power to detect a pre-specified effect size?	Sample size was demonstrated retrospectively to provide sufficient power based on the means and SD obtained. We required a sample size which would provide a power of at least 80% as calculated in Charan and Kantharia 2013.
1.b. For animal studies, include a statement about sample size estimate even if no statistical methods were used.	As in 1a, minimal sample sizes in animal studies were also determined retrospectively based on the means and SD obtained. In most cases, at the means and SD obtained, a sample size of n=5 was already sufficient to provide a power of at least 80% (Charan and Kantharia). Our actual sample sizes were 7-9, so that statistical power was satisfactory.
2. Describe inclusion/exclusion criteria if samples or animals were excluded from the analysis. Were the criteria pre-established?	Animals were excluded from the experiment if weight was reduced by >10% between sequential weightings or by >20% from initiation. These criteria were pre-estblished.
3. Were any steps taken to minimize the effects of subjective bias when allocating animals/samples to treatment (e.g. randomization procedure)? If yes, please describe.	The experimenters who measured motor and histological parameters were blinded to the treatments, which were encoded by letters and numbers not revealing the treatment.
For animal studies, include a statement about randomization even if no randomization was used.	Litters from mice born a few days apart (our breeding cages are harems of one male and 2-3 females) were pooled and pups of similar weight were divided into vehicle and 144DG11 treated arms. Hence our studies were randomized.
4.a. Were any steps taken to minimize the effects of subjective bias during group allocation or/and when assessing results (e.g. blinding of the investigator)? If yes please describe.	In the in vivo experiments, as said, experimenters were blinded to arm (treated/vehicle) allocation. In the cell-based and in vitro experiments, except for proteomics, experimenters were not blinded to group allocation. In the in silico experiments, group allocation is not applicable.
4.b. For animal studies, include a statement about blinding even if no blinding was done	In all animal studies, experimenters were blinded to group allocation.
5. For every figure, are statistical tests justified as appropriate?	Yes. The relevant statistical tests and their results were reported for each figure.
Do the data meet the assumptions of the tests (e.g., normal distribution)? Describe any methods used to assess it.	We assumed normal distribution for all groups compared. However, this assumption was not tested manily because the sample size of each group (usually n<5 biological replicates) was too small.
Is there an estimate of variation within each group of data?	Yes. In all quantitative results stanard errors (SEM, or SD) are reported to show the variation around the mean.

Is the variance similar between the groups that are being statistically compared?	Not always. However, the variance is alaways innately accounted for by all statistical tests used.

C- Reagents

6. To show that antibodies were profiled for use in the system under study (assay and species), provide a citation, catalog number and/or clone number, supplementary information or reference to an antibody validation profile. e.g., Antibodypedia (see link list at top right), 1DegreeBio (see link list at top right).	To detect LC3 we used Cell Signaling Technologies 2775 Ab, to detect HEXA we used Abcam ab230488 Ab, to detect LAMTOR4 we used Cell Signaling Technologies 13140 Ab, to detect LIMP-2 we used Abcam ab176317 Ab, to detect GAA we used Abcam ab137068 Ab, to detect GBA we used Abcam ab128879 Ab, to detect actin we used Abcam HRP-conjugated mouse Ab against beta actin ab20272, to detect LAMP1 we used Abcam H4A3 (H4A3 clone) Ab, and to detect p62 we used Cell Signaling Technology 5114 Ab. Relevant citations and information on these antibodies can be found according to catalog number in the websites of the respective suppliers.
7. Identify the source of cell lines and report if they were recently authenticated (e.g., by STR profiling) and tested for mycoplasma contamination.	NA

* for all hyperlinks, please see the table at the top right of the document

D- Animal Models

8. Report species, strain, gender, age of animals and genetic modification status where applicable. Please detail housing and husbandry conditions and the source of animals.	For in vivo work, Gbe knockin female mice homozygous for the human p.Y329S mutation were used. These Gbeys/ys mice were generated by Dr. HO Akman from Columbia University. Mouse ages ranged from 4 to 10 months according to the different experimental designs (longitudinal and end point). The genetic background of Gbeys/ys mice is C57Bl6J, also used as a wild type control. The work described was performed at the Hadassah-Hebrew University Sharett Institute SPF Animal Facility (SI-SPF) under protocols approved by the Hebrew University Institutional Animal Care and Use Committee (IACUC). Animals were housed at a density of 5 animals/cage.
9. For experiments involving live vertebrates, include a statement of compliance with ethical regulations and identify the committee(s) approving the experiments.	All experiments were ethically approved by the Hebrew-University Institutional Animal Care and Use Committees (IACUC).
10. We recommend consulting the ARRIVE guidelines (see link list at top right) (PLoS Biol. 8(6), e1000412, 2010) to ensure that other relevant aspects of animal studies are adequately reported. See author guidelines, under 'Reporting Guidelines'. See also: NIH (see link list at top right) and MRC (see link list at top right) recommendations. Please confirm compliance.	Compliance with ARRIVE guidelines is approved.

E- Human Subjects

11. Identify the committee(s) approving the study protocol.	The Hadassah Institutional Review Board (IRB).
12. Include a statement confirming that informed consent was obtained from all subjects and that the experiments conformed to the principles set out in the WMA Declaration of Helsinki and the Department of Health and Human Services Belmont Report.	Informed consent forms were obtained from all subjects (APBD patients) who contributed their skin punch biopsies for cell culturing. These informed consent forms comply with the Declaration of Helsinki and the Department of Health and Human Services Belmont Report.
13. For publication of patient photos, include a statement confirming that consent to publish was obtained.	NA
14. Report any restrictions on the availability (and/or on the use) of human data or samples.	An MDA has to be signed if human skin fibroblast cell cultures are to be shared.
15. Report the clinical trial registration number (at ClinicalTrials.gov or equivalent), where applicable.	NA
16. For phase II and III randomized controlled trials, please refer to the CONSORT flow diagram (see link list at top right) and submit the CONSORT checklist (see link list at top right) with your submission. See author guidelines, under 'Reporting Guidelines'. Please confirm you have submitted this list.	NA
17. For tumor marker prognostic studies, we recommend that you follow the REMARK reporting guidelines (see link list at top right). See author guidelines, under 'Reporting Guidelines'. Please confirm you have followed these guidelines.	NA

F- Data Accessibility

18: Provide a "Data Availability" section at the end of the Materials & Methods, listing the accession codes for data generated in this study and deposited in a public database (e.g. RNA-Seq data: Gene Expression Omnibus GSE39462, Proteomics data: PRIDE PXD000208 etc.) Please refer to our author guidelines for 'Data Deposition'.<br><br>Data deposition in a public repository is mandatory for:<br>a. Protein, DNA and RNA sequences<br>b. Macromolecular structures<br>c. Crystallographic data for small molecules<br>d. Functional genomics data<br>e. Proteomics and molecular interactions	Protomics data were deposited in the Pride repository with the dataset identifier PXD027583. The reviewer account details are Username: reviewer_pxd027583@ebi.ac.uk, Password: 9VVokm7k
19. Deposition is strongly recommended for any datasets that are central and integral to the study; please consider the journal's data policy. If no structured public repository exists for a given data type, we encourage the provision of datasets in the manuscript as a Supplementary Document (see author guidelines under 'Expanded View' or in unstructured repositories such as Dryad (see link list at top right) or Figshare (see link list at top right).	Proteomics dataset deposited in Pride.
20. Access to human clinical and genomic datasets should be provided with as few restrictions as possible while respecting ethical obligations to the patients and relevant medical and legal issues. If practically possible and compatible with the individual consent agreement used in the study, such data should be deposited in one of the major public access-controlled repositories such as dbGAP (see link list at top right) or EGA (see link list at top right).	NA
21. Computational models that are central and integral to a study should be shared without restrictions and provided in a machine-readable form. The relevant accession numbers or links should be provided. When possible, standardized format (SBML, CellML) should be used instead of scripts (e.g. MATLAB). Authors are strongly encouraged to follow the MIRIAM guidelines (see link list at top right) and deposit their model in a public database such as Biomodels (see link list at top right) or JWS Online (see link list at top right). If computer source code is provided with the paper, it should be deposited in a public repository or included in supplementary information.	The computational analysis of 144DG11 docking to LAMP1 is explained in detail in the Appendix and EV figures.

G- Dual use research of concern

22. Could your study fall under dual use research restrictions? Please check biosecurity documents (see link list at top right) and list of select agents and toxins (APHIS/CDC) (see link list at top right). According to our biosecurity guidelines, provide a statement only if it could.	NA
